# Microporous polyarylate membranes based on 3D phenolphthalein for molecular sieving

**DOI:** 10.1126/sciadv.ado7687

**Published:** 2024-08-09

**Authors:** Ayan Yao, Junjun Hou, Pengjia Dou, Jingcheng Du, Qian Sun, Ziye Song, Linghao Liu, Jian Guan, Jiangtao Liu

**Affiliations:** Department of Environmental Science and Engineering, University of Science and Technology of China, 230052, China.

## Abstract

Thin-film composite (TFC) membranes have gradually replaced some traditional technologies in the extraction, separation, and concentration of high value-added pharmaceutical ingredients due to their controllable microstructure. Nevertheless, devising solvent-stable, scalable TFC membranes with high permeance and efficient molecule selectivity is urgently needed to improve the separation efficiency in the separation process. Here, we propose phenolphthalein, a commercial acid-base indicator, as an economical monomer for optimizing the micropore structure of selective layers with thickness down to 30 nanometers formed by in situ interfacial reactions. Molecular dynamics simulations indicate that the polyarylate membranes prepared using three-dimensional phenolphthalein monomers exhibit tunable microporosity and higher pore interconnectivity. Moreover, the TFC membranes show a high methanol permeance (9.9 ± 0.1 liters per square meter per hour per bar) and small molecular weight cutoff (≈289 daltons) for organic micropollutants in organic solvent systems. The polyarylate membranes exhibit higher mechanical strength (2.4 versus 0.8 gigapascals) compared to the traditional polyamide membrane.

## Teaser

The prepared polyarylate membranes exhibit tunable microporosity and higher pore interconnectivity.

## INTRODUCTION

The syntheses of high value-added active pharmaceutical ingredients usually require multistep molecular separation of target compounds in different organic solvents (*1*–*3*). Traditional liquid separation processes, such as distillation or evaporation, require considerable cost and energy consumption to purify high value-added solutes from organic solvents (*4*, *5*). The application of advanced membrane technology in separation, purification, and concentration processes is an effective way to increase economic benefits or reduce investment in the pharmaceutical industry (*6*–*9*). Consequently, membrane technology, which has garnered global attention, is widely recognized as the most promising major production technology in the 21st century.

In the process of membrane separation, organic solvent nanofiltration (OSN) is considered an advanced technology due to its simplicity, energy efficiency, high efficacy, and enhanced safety features. Furthermore, it effectively circumvents phase changes and secondary contamination during drug separation and purification processes, particularly suitable for treating heat-sensitive substances and bioactive compounds (*8*, *10*, *11*). Thin-film composite (TFC) membranes, a type of classical OSN membrane prepared by interfacial polymerization (IP) on the ultrafiltration membrane, dominate the OSN market because of their industrially scalable, low-cost, and simple operation (*4*, *12*). To further improve membrane separation efficiency, designing polymer membrane structures at the molecular level to provide larger interconnected microporosity is a promising strategy. Early reports demonstrated that highly permeable polyamide (PA) TFC membranes could be successfully produced by IP of three-dimensional (3D) reactive monomers, e.g., triptycene-1,3,6,8-tetraacetyl chloride reacted with *m*-phenylene diamine (MPD) proposed for use in OSN (*13*). Similarly, Hu and colleagues (*14*) reported polyarylate (PAR) microporous TFC OSN membrane of 3D BIPOL-based monomer reacted with trimesoyl chloride (TMC) by IP for OSN applications. Back in 2016, Livingston and colleagues (*15*) demonstrated that IP-based PAR TFC membranes could be successfully produced from 3D monomers, and experimental results showed that the synthetic 3D cross-linked PAR microporous polymers exhibited good separation properties in organic solvents. However, despite the resulting membranes demonstrating high solvent permeances due to their microporous structure, the synthesis process for these 3D phenol structures is complex and costly, thereby limiting their potential for commercial OSN applications. Therefore, the development of materials to prepare nanofiltration membranes with high economic efficiency remains a challenge.

The ideal membrane material should have high permeance and rejection and can handle a large number of solvents with a feasible membrane area in a feasible time. Therefore, choosing the appropriate reaction monomer plays a crucial role. Phenolphthalein (PN) emerges as a viable choice due to its economical nature and widespread commercial availability (*16*–*18*). Notably, PN is widely used as an important building block for condensation polymers due to its bulky pendent groups and a non-coplanar twisted structure. Various PN-based polymers have been synthesized, including poly(aryl ether ketone) (*19*, *20*), poly(aryl ether sulfone) (*16*, *21*), PARs (*22*), and poly(arylene ether nitrile) (*23*), which have excellent mechanical property and thermal and environmental stability. These features position PN as a promising monomer for enhancing the structural rigidity and microporosity of TFC membranes for OSN. Some research has described gas separation using polymer membranes prepared from different PN monomers, such as poly(aryl ether ketone) (*24*–*26*), polyimide (*27*, *28*), polybenzoxazole (*28*), poly(aryl ether sulfone) (*25*), and PARs (*15*). A substantial enhancement in gas permselectivity has been achieved for the PN-derived polymeric membranes compared to traditional polymer membranes. However, PN-based polymers have rarely been explored in liquid separation such as nanofiltration of organic solvents.

Here, we report the successful fabrication of highly permeable and selective OSN membranes via IP between TMC and PN building blocks with a non-coplanar structure, which results from the high microporosity and interconnectivity of the PAR TFC membranes. The pore size and structure of TFC membranes could be tuned by substituting side-chain groups (such as methyl and isopropyl groups) near phenolic hydroxyl groups. To demonstrate the origin of high performance using PN-based TFC membranes as OSN membranes, molecular dynamics (MD) simulations were performed to construct realistic structural models. Furthermore, we systematically investigated the effects of critical synthetic parameters of PAR TFC membranes, such as the concentration and species of PN monomers and the amount of NaOH, to elucidate their effects on the nanofiltration performance of the resulting polymer membranes. The results show that the side-chain groups near the PN hydroxyl group make the surface of the membrane more hydrophobic, and the pore size of the membrane also changes, mainly reflected in the enlargement of the pore size. Accordingly, this work provides a strategy for using the interfacial reaction of 3D monomers to form high-performance and structurally tunable TFC membranes for OSN applications.

## RESULTS

### Construction and characterization of PAR membranes

**Fig. 1.**
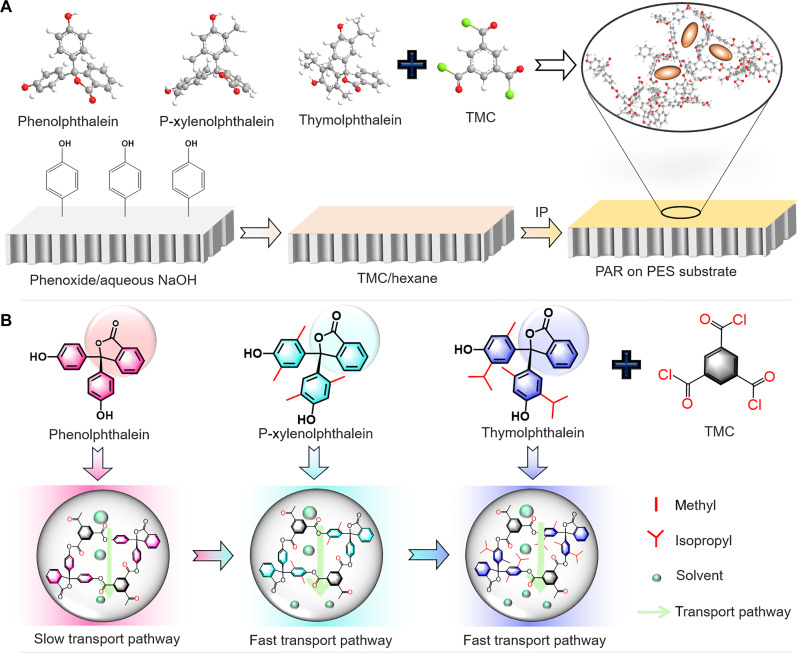
Preparation and characterization of PAR membranes with three phenolphthalein monomers. (**A**) The reaction between hydroxyl groups of PN and reactive acyl chloride groups of TMC. The dihydroxy monomers were used: PN, PXN, and TN. Atoms color code: C, gray; H, white; O, red; Cl, green. The bottom diagram shows a surface-triggered IP. The illustration in the black circle shows the microporous structure of the resulting PAR membrane. (**B**) Proposed pore structure of PAR membranes, and schematic representation of the OSN process through the PAR membranes. The PAR membranes with contorted solvent channels, slow transport pathway of PAR-PN membrane without branch chain, and fast transport pathway of PAR-PXN and PAR-TN membranes with methyl and isopropyl groups.

IP strategy was developed for the formation of large-area and defect-free PAR membranes for OSN (fig. S1). In detail, the PAR membranes were fabricated on a free hexane/water interface by reacting TMC with three different phenols, PN, P-xylenolphthalein (PXN), and thymolphthalein (TN) using NaOH as the catalyst. We used contorted branched phenols including PXN and TN to form PAR nanofilms with enhanced free volume and porosity and selected PN with unbranched structures as controls (Fig. 1A and fig. S2). The corresponding highly cross-linked PAR networks were named PAR-PN, PAR-PXN, and PAR-TN, respectively. As the monomer size increases from PN to TN, the occupied volume and free volume of PAR-PN, PAR-PXN, and PAR-TN after cross-linking increase continuously due to the increase of the monomer carbon chain, resulting in increased molecular spacing (table S1). Previous studies have shown that the high microporosity of polymer networks is essential for the preparation of nanofiltration membranes with high permeance (*15*, *29*–*31*). Therefore, we hypothesize that monomer branch chains (methyl and isopropyl groups) of PXN and TN monomers with branch chains (methyl and isopropyl groups) enhance the microporosity of PAR membranes and create more solvent transport channels (Fig. 1B), which are conducive to improving the membrane permeance.

**Fig. 2.**
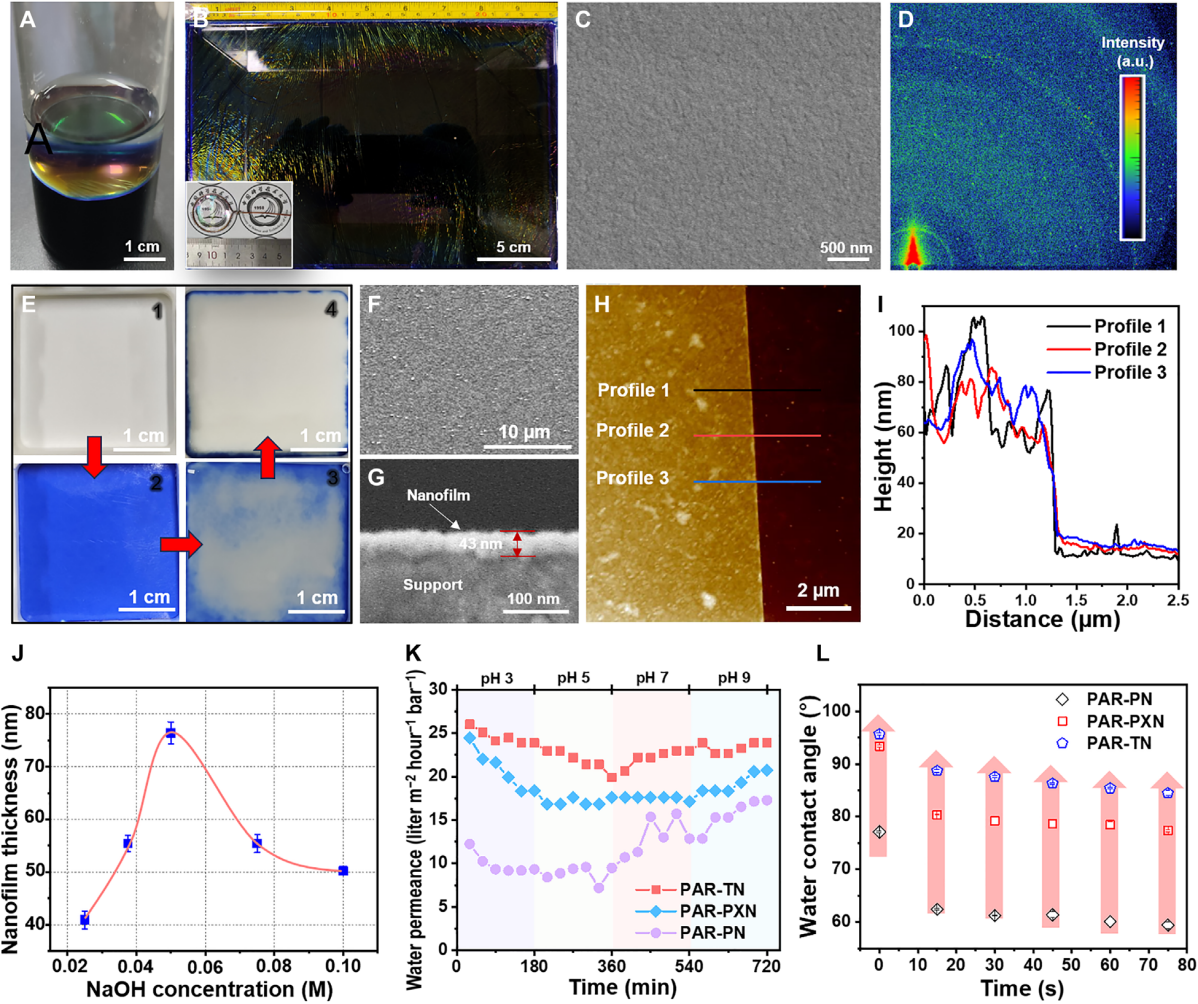
Morphology and characteristics of PAR nanofilms prepared via interfacial polymerization. (**A**) Photographs of the freestanding PAR-TN nanofilm formed at the free organic/water interface. (**B**) Photograph of large-size (16.6 cm by 25 cm) freestanding nanofilm, floating in the water and captured by a wire loop (inset). (**C**) Scanning electron microscopy (SEM) and (**D**) grazing-incidence wide-angle x-ray scattering 2D images of PAR nanofilms on a SiO_2_/Si substrate. (**E**) The fabrication processes of the PAR TFC membranes based on polyether sulfone (PES) support. Among them, (i) represents the PES substrate, (ii) represents the surface of the membrane after immersion in TN solution of sodium hydroxide, (iii) represents the surface of the membrane during the reaction, and (iv) represents the surface of the prepared TFC membrane. SEM images of the surface (**F**) and cross section (**G**) of the PAR TFC membranes. Atomic force microscopy image (**H**) and corresponding height profile (**I**) of a PAR-TN membrane on top of a silicon wafer. (**J**) Thickness of the nanofilm with different NaOH concentrations. (**K**) Influence of feed solution pH in water permeance of PAR membranes. (**L**) Water contact angles over a wetting time of 75 s.

The freestanding nanofilms comprising highly cross-linked PAR networks can be obtained through the interfacial reaction between the hydroxyl groups of PN and highly reactive acyl chloride groups of TMC (Fig. 2A). The flexible and tough PAR nanofilms show no visible tear or fragmentation after being shaken violently when scaling the nanofilm up to a larger area and could be isolated by a copper wire loop (fig. S3 and movies S1 to S3). Figure 2B presents a freestanding nanofilm of large size (16.6 cm by 25 cm) displayed at an aqueous-air surface while remaining macroscopically defect-free. The scanning electron microscopy (SEM) image of the PAR nanofilm transferred on a porous polyether sulfone (PES) substrate shows a smooth, continuous, and defect-free microstructure (Fig. 2C). In addition, grazing-incidence wide-angle x-ray scattering (GIWAXS) was used to investigate the orientation of PAR nanofilms. The fuzzy Debye-Scherrer rings can be seen in the GIWAXS pattern in Fig. 2D and figs. S4 and S5, indicating the random orientation of the ultrathin PAR nanofilms (*32*–*34*). The chemical composition of the three PAR films is very similar, but the different branch chains make the arrangement of the corresponding polymer network notably different.

Considering the scale-up potential, membranes of large size are suitable for use in plate-and-frame industrial applications (*35*). A series of PAR composite membranes were directly prepared on PES substrate resistant to organic solvent (fig. S6) and verified by SEM images. PN, as a color indicator, can be used to track the formation of membranes. The disappearance of the color on the PES substrate confirms that the phenol monomer reacts with TMC completely, thus transmitting the signal of the formation of the PAR membranes (Fig. 2E and fig. S7). The same condition produced a continuous dense and smooth nanofilm on top surface of a PES support as evidenced by the SEM analysis (Fig. 2F). The formation of a smooth membrane surface is attributed to the non-coplanar structure of PN macromolecules, which results in their slower diffusion compared with conventional amine monomers [such as MPD and piperazine (PIP)] and makes them more susceptible to steric hindrance of the formed continuous nanofilms (*14*, *15*, *31*).

Nevertheless, high-permeance PAR membranes are desired to be prepared, mainly by preparing a thin selective layer and forming a “ridge and valley” topography on the TFC membrane surface (i.e., improving the effective filtration area) (*36*, *37*). This could be achieved by regulating the amount of NaOH and the concentration of monomer (fig. S8). The SEM results reveal that at a catalyst concentration of 0.075 M, the membrane surface exhibits a slight “ridge and valley” morphology that promotes enhanced permeance. The atomic force microscopy (AFM) analysis further verified the results (figs. S9 and S10). By adjusting the NaOH concentration while maintaining the reaction monomer concentration, a maximum roughness of 86.5 nm is achieved at the NaOH concentration of 0.075 M. Moreover, the cross-sectional SEM images revealed that the PAR selective layer was attached to the top surface of PES support with an identifiable boundary layer, and the thickness of PAR-TN nanofilms was 43 nm (Fig. 2G). TEM cross-sectional images show nearly the same thickness as the selective layer (fig. S11). In addition, we also found that the active layer of the composite membranes is of similar thickness to that of the equivalent freestanding nanofilm (Fig. 2, G to I, and fig. S11). The PAR selective layer thickness could be reliably tuned by varying the NaOH and monomer concentrations (figs. S12 and S13).

The chemical structure of the PAR nanofilms as PAR was validated using attenuated total reflection–Fourier transform infrared (ATR-FTIR) (fig. S14). The characteristic vibration of the C═O group (1733 cm^−1^) revealed the formation of ester bonds via IP reaction between the hydroxyl groups of phenol and the acyl chloride groups of TMC. X-ray photoelectron spectroscopy (XPS) spectra were used to further investigate the chemical compositions on the nanofilm surface. Figure S15 depicts the XPS spectra of the PES substrate and PAR TFC membranes, while fig. S15B summarizes the atomic composition calculated from XPS spectra. Compared with the PES support, the oxygen content of PAR TFC membranes increases from 23.8 to 25.8%, which not only proves the formation of the PAR network but also shows that the cross-linking degree of three membranes follows the trend of PAR-PN > PAR-PXN > PAR-TN. Because the IP between TMC and PN is esterification, the C/O ratio can be used to qualitatively compare the relative changes of cross-linking degree (*29*). As shown in fig. S16, the C/O ratio of the PAR membrane shows volcanic-type curves with increasing NaOH concentration, and the ratio declines after reaching a maximum value at 0.05 M NaOH. The initial increase indicates that PN exposed more deprotonated reaction sites with the increase of NaOH concentration in the range of 0.0125 to 0.05 M, resulting in more cross-linking and thicker membrane. The subsequent decline indicates that a higher NaOH concentration caused the disruption of the PAR-selective layer [i.e., lower cross-linking degree and thickness are formed (Fig. 2J and fig. S17)]. This is consistent with the SEM results of the membrane cross section above. Notably, the permeance of PAR membranes could be adjusted by controlling the pH of the feed solution. For instance, the pH of the feed solution increases from 3 to 9 (Fig. 2K), and the permeance of the membranes shows a trend of decreasing first and then increasing. This observation is closely related to the reduction of the cross-linking degree of PAR networks at higher pH, that is, the excess of OH^−^ ions can gradually hydrolyze the ester bonds. XPS demonstrated that the C/O value decreased as the pH value of the feed solution increased from 5 to 9 (fig. S17), indicating a decreased cross-linking degree of membranes.

**Fig. 3.**
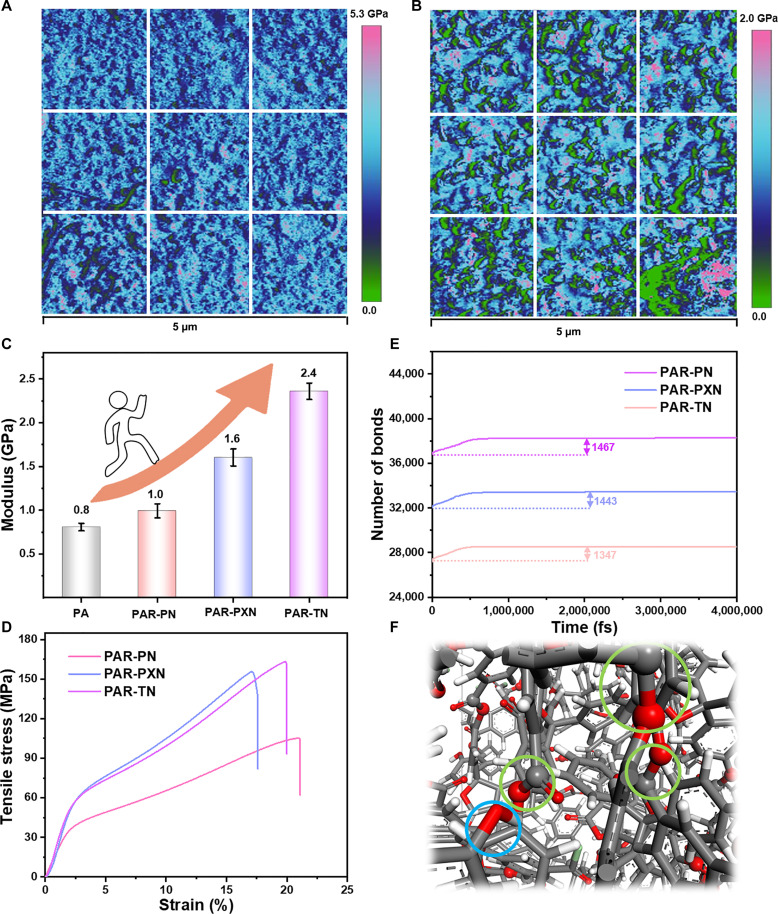
Comparison of mechanical properties of forming PAR and PA membranes. Young’s modulus mapping of (**A**) PAR-TN and (**B**) PA membranes tested using the PFQNM method. (**C**) Young’s modulus histogram of prepared PAR and PA membranes. (**D**) Stress-strain curves of the PAR-PN, PAR-PXN, and PAR-TN membranes tested using a microcomputer-controlled electronic tensile testing machine. (**E**) The number of ester bond formations in PAR-PN, PAR-PXN, and PAR-TN membranes with time was simulated by molecular dynamics. (**F**) The bonding cross-linking details of PAR membranes. The whole system is shown in a stick-like model. Blue circle: unreacted phenol hydroxyl group; green circle: ester group produced by the reaction.

Ordinarily, the pore surface chemistry of the PAR membrane can achieve effective control of the membrane pores and surfaces by pre-integrating different reaction groups (*30*, *38*). This concept was verified using branched PN, namely, PXN and TN, grafting hydrophobic methyl and isopropyl groups in the vicinity of the −OH. As shown in Fig. 2L and fig. S18, the contact angles of the PAR-PN, PAR-PXN, and PAR-TN membranes are 77.1°, 93.4°, and 95.7°, respectively. Obviously, the presence of alkyl is a decisive factor in the transition from hydrophilic to hydrophobicity of the PAR membrane (fig. S19). Moreover, the more hydrophobic surface creates the potential for rapid transport of organic solvents, which is attributed to expanded alkyl groups that enhance solvent-membrane interactions (*39*). Thermal analysis shows that all PAR membranes decompose at temperatures exceeding 400°C in a nitrogen atmosphere (fig. S20), demonstrating the exceptional thermal stability of the formed membranes. In addition, a small turning peak is clearly visible on the DTG curve of the PAR membranes, which is believed to be caused by the thermal-induced loss of the PN side group from the polymer main chain. Furthermore, such PAR membranes, with Young’s modulus in the range of 1.0 and 2.4 GPa depending on the PN unit (Fig. 3, A and B, and fig. S21), exhibit remarkable mechanical strength. It should be noted that the average Young’s modulus of PAR-PXN and PAR-TN membranes is obviously higher than that of PAR-PN membranes because of the presence of branch chains. In addition, the traditional PA membrane only has a performance value of 0.8 GPa, which is notably lower than that of PAR-PN (1.0 GPa), PAR-PXN (1.6 GPa), and PAR-TN (2.4 GPa) membranes (Fig. 3C and table S2). The difference in mechanical properties (elongation at break and tensile strength) between the PA membrane and the three PAR membranes could also be seen from the stress-strain curves shown in Fig. 3D and fig. S22. It is worth noting that although the absolute values obtained by stretching and the peak force quantitative nanomechanical mapping (PFQNM) method are slightly different due to the difference different membrane preparation methods and test principles, both methods confirm that Young’s modulus of the PAR-PXN and PAR-TN membranes is higher than that of the PAR-PN membrane. This phenomenon is further explained by the simulation results, that is, the ester bond number of PAR-TN and PAR-PXN networks is greater than that of PAR-PN networks after the system is stabilized (Figs. 3, E and F). Therefore, the mechanical strength of the three PAR membranes follows PAR-TN > PAR-PXN > PAR-PN.

### Separation performance of polyarylate TFC membranes

**Fig. 4.**
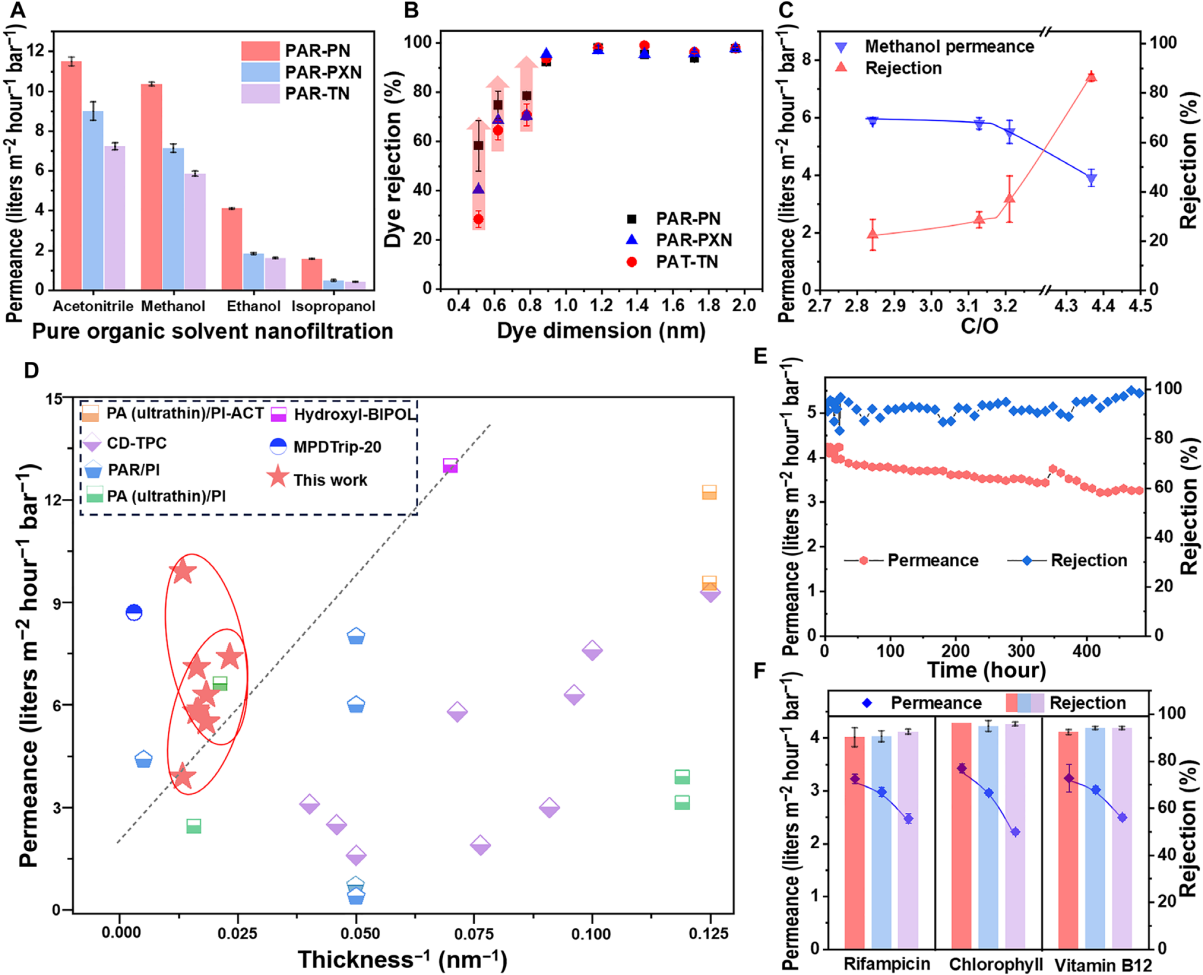
Organic solvent nanofiltration performances of PAR membranes. (**A**) Pure solvent permeances of acetonitrile, methanol, ethanol, and isopropyl alcohol through PAR nanofilms composite membranes prepared on PES supports. (**B**) Rejection properties of small cavity (PAR-PN), medium cavity (PAR-PXN), and large cavity (PAR-TN) membranes for dye molecules of different sizes. (**C**) Methanol permeance and methyl orange rejection of the nanofilms made from PAR-PXN with varying C/O ratio (i.e., cross-linking degree). (**D**) Separation performance of prepared PAR membranes in comparison with the recently reported nanofiltration membranes. Notes: OSN was conducted in permeance for methanol versus the reciprocal membrane thickness for PAR nanofilms. Previously reported OSN membranes are included for comparison, which consists of PA and PAR membranes. Based on the reported literature value, the dashed line shows the upper bound of the solvent permeance (permeance per unit thickness). (**E**) Methanol permeance and VB12 rejection of PAR-TN membrane during continuous filtration for 480 hours. (**F**) Comparison of separation performance of three kinds of PAR membranes for chlorophyll, rifampicin, and vitamin B12. The red, blue, and purple columns represent the PAR-TN, PAR-PXN, and PAR-PN membranes, respectively.

The effect of PN monomers with different branch chains on the solvent permeance of the composite membrane is shown in Fig. 4A. For all PAR membranes, it should be noted that methanol and acetonitrile, with low viscosity (η), give higher permeance (table S3), followed by ethanol and, lastly, isopropanol. The permeance of three alcohols follows the order of methanol > ethanol > isopropanol, which is also influenced by their molecular weight, kinetic diameter, and relative polarity (*40*–*42*). Furthermore, we found that the permeance of PAR-PN membranes was notably lower than that of PAR-PXN and PAR-TN, regardless of the solvent used. This is because the size of PXN and TN molecules containing alkyl side chains is larger than the PN molecule, which reduces the diffusion rate of the monomer toward the interface during IP, thus forming a thin and porous selective layer (fig. S23). The diffusion experiments of three PN molecules during the reaction confirmed our conjecture (fig. S24). Moreover, PAR membranes exhibit outstanding permeance stability in several typical organic solvents such as methanol, ethanol, and isopropanol (fig. S25). Separation tests of dyes with different molecular weights were carried out to explore the density of the three PAR TFC membranes. The rejection data of the three PAR membranes prepared under identical conditions followed the order of PAR-PN > PAR-PXN > PAR-TN membranes (Fig. 4B). This shows that the rejection change corresponds well to the size of the PN chain, mainly manifested as a decreasing trend of intercession with the increase of alkyl chain. Therefore, we concluded that the microporosity of the cross-linked PAR network is enhanced because of the presence of the alkyl chain of the PN monomer so the rejection reduction is accompanied by the improvement of the permeance (*43*, *44*).

In addition, we propose that the cross-linking degree and thickness of the PAR network ultimately determine the permeance and rejection of the formed membranes. A series of PAR TFC membranes were prepared using PN with different alkyl chains for OSN to verify the above statement (table S4). We systematically explored the effects of crucial synthetic parameters, including the amount of NaOH and the concentration of monomers, to elucidate their effects on the OSN performance of the obtained PAR membranes. The molecular separation properties of these membranes were evaluated by separating a series of dyes dissolved in methanol (fig. S26). Figure S27 shows that at an appropriate concentration of NaOH (e.g., 0.05 M), the PAR-PN membrane exhibits satisfactory rejection, which is attributed to the adequate deprotonation of the −OH group of PN (that is, the PN is adequately reacted) to obtain a thicker PAR selective layer. When the concentration of NaOH increases to 0.075 M, the membrane permeance increases substantially (from 5.4 to 9.9 liters m^−2^ hour^−1^ bar^−1^), although the rejection to Direct red 23 decreases slightly (from 98.1 to 93.2%), indicating that excessive NaOH would lead to a decrease in the cross-linking degree of the polymer network, resulting in an ultrathin PAR selective layer. This is consistent with the SEM results in fig. S12. The TMC concentration also plays a crucial role in the formation of the selective layer and in the OSN performance of the resulting PAR membrane. As shown in fig. S28, when the concentrations of PXN and NaOH are 0.2% w/v and 0.025 M, respectively, the rejection of Neutral Red (NR; 289 g mol^−1^) by PAR-PXN membrane increases from 49 to 94.4% as the TMC concentration increases from 0.05 to 0.3% w/v. This enhanced rejection of small molecular dyes with the increase of TMC concentration is mainly due to the higher degree of cross-linking of the polymer network (Fig. 4C) with the increase of TMC concentration, which is consistent with the SEM results (fig. S12). In addition, PN monomers concentration also plays an irreplaceable role in the selective layer formation and ensuring OSN performance (fig. S29). Anyway, we assert that the microporosity arising from the non-coplanar contorted structure of PN constitutes the fundamental determinant for ensuring the exceptionally high permeance of the PAR membrane. The concentrations of catalyst and reaction monomers could control the thickness of the membrane by adjusting the cross-linking degree of the PAR network to maintain the high rejection of the PAR membrane.

Figure 4D summarizes the OSN performance comparison between the formed PAR membrane and the previously reported nanofiltration membranes, including PA and PAR membranes, which is mainly reflected by the relationship between methanol permeance and reciprocal thickness (table S5). On the basis of previous reports, a gray dashed line was added to the diagram, with a slope indicating permeance per unit thickness to show the potential of membranes in the process of solvent transport (*30*). Substantially, only polymer membranes based on contorted monomers are more likely to be permeable above the dotted line, while traditional PA membranes and newer membranes, such as cyclodextrin-based membranes (*45*), have fluxes well below the dotted line. Most of the PN-based PAR membranes are far outperform other reported membranes. Compared with membranes based on Trip and BIPOL monomers (*13*, *14*), the polymerization reaction between monomers containing “3 + 2” functional groups in PN-based membranes not only ensures that the membranes have enough cross-linking degree to achieve efficient separation but also forms a polymer network with ultrahigh microporosity to provide a fast solvent transport channel. In addition, the OSN performance of the prepared PAR membranes was compared with the reported advanced membranes (table S6), showing the trade-off between permeance and rejection, and also indicating that the pore aperture of the PAR membrane can be rationally regulated according to the size of the target molecule to achieve the multiple separation purpose. The durability of OSN performance determines the industrial perspectives of the membrane. Therefore, a long-term filtration test was carried out to verify the operational durability of the prepared PAR membranes. As shown in Fig. 4E, the rejection of vitamin B12 remained unexpectedly constant over 480 hours of operation, while the methanol permeance decreased slightly but eventually stabilized at 3.3 liters m^−2^ hour^−1^ bar^−1^, and the stably operated duration of the PAR membrane is much higher than that of other (fig. S30). Notably, the decrease in permeance is attributed to the flow-induced compaction and the intricate polymer-solvent interactions caused by the flow of methanol through the PAR membrane pores (*46*). Moreover, PAR nanofilm showed good tolerance to various organic solvents (fig. S31).

This creates the potential for applying PAR nanofiltration membranes in high value-added pharmaceutical separations requiring accurate molecular sieving, exemplified here by the enrichment of important raw drugs (*9*, *47*–*49*). In this regard, the selection of three PAR membranes for separation testing of raw drugs with different functions and structures demonstrates the competence of the formed membranes to handle a wide range of drugs. The PAR membranes show rejections of chlorophyll, rifampicin, and vitamin B12 greater than 92% while maintaining a high ethanol permeance (Fig. 4F and fig. S32). In general, the enrichment of drug extract is one of the important processes in the pharmaceutical industry. We preferentially selected rifampicin as a model drug to explore the application potential of the formed membranes in the enrichment of heat-sensitive drugs. Figure S33 presents the experimental results of the concentration of rifampicin/ethanol [100 parts per million (ppm)] for 18 hours using the PAR-TN membrane. The ultraviolet (UV) spectral results showed that rifampicin could be efficiently rejected from ethanol by the PAR-TN membrane. Initially, the permeance of the PAR-TN membrane remained around 2.2 liter m^−2^ hour^−1^ bar^−1^. After 17 hours, rifampicin in ethanol could be enriched to about 213 ppm, double that of the raw feed concentration. In addition, typical experiments using PAR-PXN membranes to enrich chlorophyll from methanol were also performed (fig. S34). Although the permeance of the membrane decreased slightly with the advance of time, the high permeance of 3.2 liters m^−2^ hour^−1^ bar^−1^ was maintained and the rejection was close to 100% after 10 hours, while the high purity alcohol could be recovered on the permeate side. Unlike predecessors who assessed OSN performance only by rejecting dyes, this work consciously confirmed the usefulness of PAR membranes in the purification of high value-added pharmaceutical molecules.

### High OSN performance mechanism of the PAR membrane

We selected commercial PN instead of conventional aqueous monomers (such as MPD and PIP) because it contains a larger molecular size and tunable reactive hydroxyl (−OH) group, which can better regulate the degree of polymerization. In addition, the 3D molecular structure of PN is more contorted, and the molecular non-coplanarity is strong, which can enhance the free volume and interconnected voids in the selective layer structure. These characteristics of PN may be crucial factors in achieving high permeance and accurate molecule purification.

**Fig. 5.**
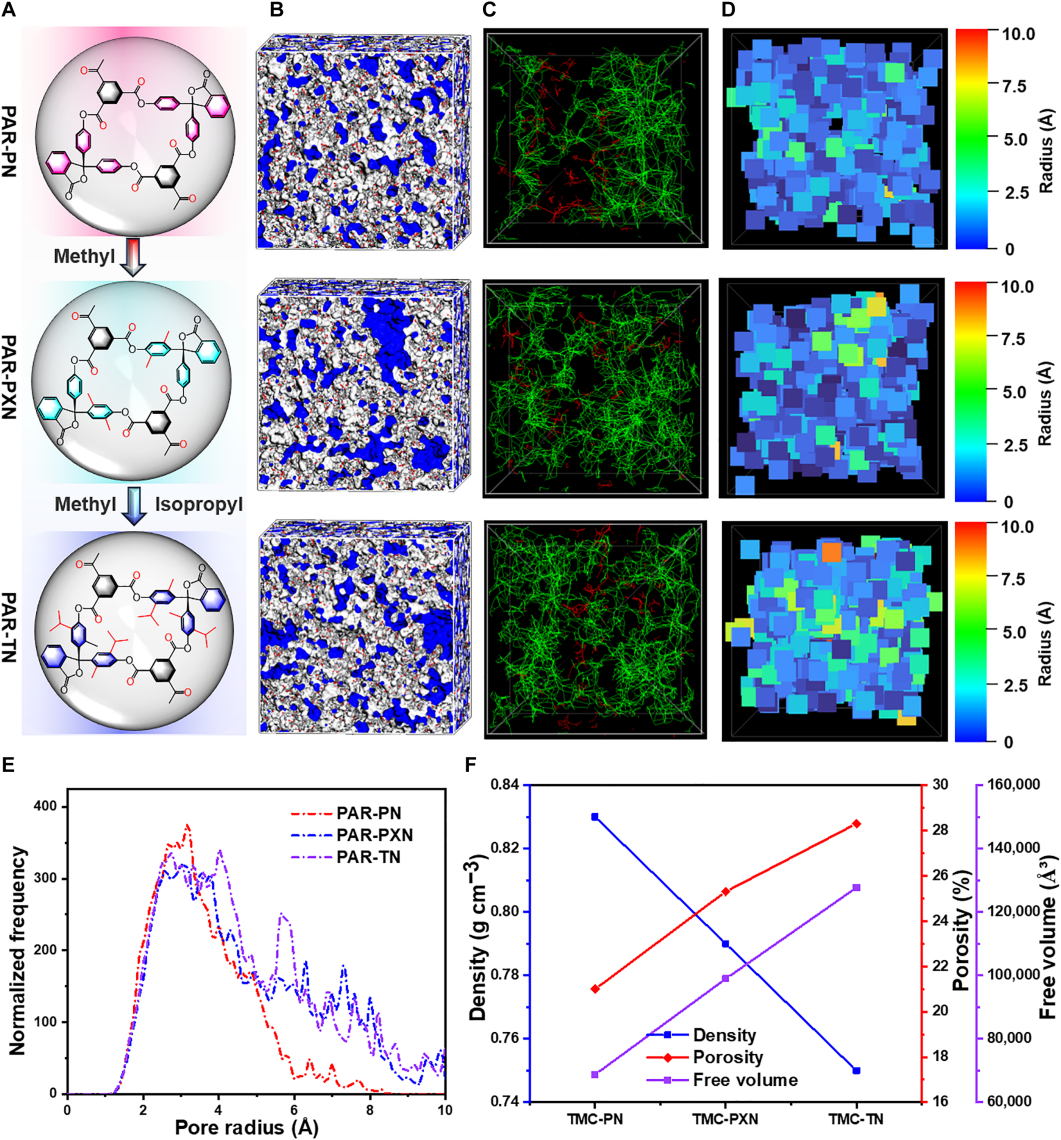
Structural analysis of polyarylate network models. (**A**) Pore model diagram of PAR networks with different side-chain groups. (**B**) 3D view of an amorphous cell containing non-coplanar contorted PAR networks. The gray and blue shades indicate the free volume detected by a probe with a radius of 0.6 Å. (**C**) Interconnected (green) and disconnected (red) voids in the PAR network with respect to a probe of 0.6-Å radius, which could be distributed across the rigid models. These images show the difference between PAR-PN, PAR-PXN, and PAR-TN. (**D**) The corresponding voids of the PAR network colored with respect to the pore radius are shown. (**E**) The simulated pore size distribution of the PAR network system. (**F**) The simulated density, free volume, and porosity value of the PAR membranes.

In addition, this hypothesis that the influence of different PN monomers on the pore structure of the PAR membranes after cross-linking is mainly due to the alkyl branch chain near the hydroxyl group needs to be verified (Fig. 5A). Therefore, the pore structure of the prepared PAR membranes was investigated by the realistic structure model generated from MD simulation based on PACKMOL software with the generation Amber force field. All the MD simulations were performed by using the LAMMPS package (*50*). Zeo++ (*51*) was used to analyze the voids of three PAR membranes with a probe radius of 0.6 Å, including void space, pore size distribution, and the interconnectivity of void space. All three PN molecules have 3D contorted structures, and after reacting with TMC, the PN building blocks are kept in non-coplanar orientation by the PAR network, which enhances the interconnection of holes in the PAR network membrane (fig. S35). Furthermore, the fractional free volume values of the three PAR membranes exhibit an upward trend (Fig. 5B and table S1), which further indicates that the microporosity of the three PAR networks followed the order PAR-TN > PAR-PXN > PAR-PN. Figure 5C presents interconnected and disconnected voids of PAR-TN, PAR-PXN, and PAR-PN networks. All PAR-PN, PAR-PXN, and PAR-TN membranes show high porosity and mostly interconnected voids, illustrating that the results of using non-coplanar twisted structures produce membranes with enhanced microporosity and interconnectivity. Figure 5D further displays the voids in the polymer network, which are colored according to the maximum probe diameter that could be inserted. It could be seen that the voids of PAR-PXN and PAR-TN membranes are larger than those in the PAR-PN membrane, indicating that the PAR membrane containing an alkyl branched chain contributes to the increased microporosity. A comparison of the pore connectivity of simulated PAR-PN, PAR-PXN, and PAR-TN membranes under different probe sizes was also performed in figs. S36 and S37. In sharp contrast, PAR-PXN and PAR-TN membranes exhibited higher porosity and better pore connectivity than PAR-PN when using large 0.7- and 0.8-Å probes, demonstrating that the alkyl branched chain substantially increases microporosity and connectivity. The pore size distribution of PN-based PAR networks obtained by these simulations showed a similar trend (Fig. 5E). The larger the aperture, the lower the rejection, which is an indisputable fact. As the length of the alkyl branch chain increases, the aperture of the simulation increases exactly as we assume from the OSN test results. Figure S38 shows the pore size distribution of each PAR network from the above simulation results, with the largest pore sizes of PAR-PN, PAR-PXN, and PAR-TN being 8, 11, and 12 Å, respectively. In addition, the real pore size distribution of the PAR membranes has been also measured with CO_2_ as the test atmosphere. As shown in fig. S39, the largest pore sizes of the PAR-PN, PAR-PXN, and PAR-TN nanofilms are 8.7, 8.9, and 9.1 Å, respectively. Obviously, the largest pore size obtained by molecular simulation matches well with the real pore size of PAR membranes measured by CO_2_ adsorption, which proves the reliability of the simulation results. By contrast, the mean pore size of the PAR networks measured by CO_2_ adsorption is slightly larger than the simulated mean pore sizes (fig. S39), which indicates that the true pore diameter is underestimated in the simulated structure due to the ignored organic solvent environment in the simulation. A similar situation has been observed in previous reports, which is attributed to the swelling of the membrane in an organic solvent (*14*, *15*, *30*). Furthermore, by visualizing the porosity, density, and free volume of the three PAR networks, it is found that PAR-PXN and PAR-TN networks have relatively small densities but retain higher porosity and free volume than PAR-PN (Fig. 5F and table S1). This further illustrates that the PAR membranes have satisfactory OSN performance while also having good mechanical properties.

## DISCUSSION

In summary, we present a series of ultrathin PAR membranes with interconnected voids constructed by contorted 3D PN for OSN. These membranes demonstrated better separation performance than commercially available and previously reported membranes in purifying molecular contaminants and high value-added active pharmaceutical components from organic solvents. The separation experiments of different dye molecules and MD simulation confirmed that the free volume of the PAR networks could be tuned by using different branch chains of PN monomers. Additional advantages of PN-based PAR membranes include efficient molecule rejection (molecular weight cutoff of 289 g mol^−1^) and faster transport of organic solvents (9.9 liters m^−2^ hour^−1^ bar^−1^). In addition, both the PES ultrafiltration membrane used as a support and the reaction monomer used to form the PAR network have already been commercialized, which shows the potential to scale up. This work demonstrated that rational molecular design could lead to the development of membrane materials with high performance to truly enable molecular separation in industrial settings.

## MATERIALS AND METHODS

### Materials

PES substrates were obtained from GMT GmbH, Rheinfelden, Germany, and used as supports for nanofilms during performance testing. PN (99%) and TN (99%) were provided by the Energy Chemical. PXN (99%) was provided by the Macklin. TMC and sodium hydroxide (NaOH) were purchased from Aladdin Reagent Co. Ltd. (Shanghai, China). Direct red 80, Brilliant blue R, Methyl orange, Congo red, NR, Crystal violet, vitamin B12, and Direct red 23 from Sigma-Aldrich were used as the micropollutant models at a concentration of 30 ppm in the solvent during OSN tests. The organic solvents (such as methanol, acetonitrile, isopropanol, and ethanol) of analytical grade were used as received and were purchased from the Sinopharm Chemical Reagent Co., Ltd. Petri dishes and glass sheets were purchased from Shanghai Glass Instrument Factory (Shanghai, China). Deionized (DI) water was used throughout this study.

### Fabrication of freestanding polyarylate nanofilms

Freestanding PAR nanofilms were fabricated at the water/*n*-hexane interface by IP. Phenol monomers were first dissolved in aqueous basic NaOH solutions with varied concentrations of PN (0.2% w/v), TN (0.2% w/v), and PXN (0.2% w/v). Then, the TMC monomer was dissolved in *n*-hexane with a concentration of 0.05% w/v. As shown in fig. S1, the phenol solution was first poured into a glass petri dish as the aqueous phase, and then, TMC solution was slowly added onto the top surface as the organic phase, leaving a stable water/organic interface. Afterward, the petri dish was covered to avoid airflow and kept in a stable condition. After the interfacial reaction at room temperature for 20 min, the formed polymer nanofilm was scooped out of the interface with a wire loop and washed with DI water (fig. S2).

### Fabrication of polyarylate TFC membranes.

PN-based TFC membranes were prepared by IP directly on PES ultrafiltration supports using different monomers for OSN. IP to form PAR membranes was performed by exposing the surface of the porous substrate to an aqueous basic solution of sodium phenoxide, including varied concentrations of PN, TN, and PXN for 5 min. The phenoxide-loaded support membranes were subjected to a paper towel to remove excess aqueous solution. The membranes were then exposed to TMC in hexane for 10 min. The formed membranes were withdrawn from the hexane solution, dried in air, and cured in a ventilated oven at 60°C for 3 min to complete cross-linking.

### Scanning electron microscopy

The surface and cross section of the membranes were inspected by scanning electron microscope (SEM; ZEISS Sigma 300). A dry sample for cross-sectional images was obtained by breaking the membrane with a sharp scalpel following immersion in liquid N_2_. Before tests, samples were sputter coated with Pt/Pd alloy to improve conductivity using an auto fine coater operating at 30 mA.

### Atomic force microscopy

The surface microstructures of the membranes were analyzed by atomic force microscope (AFM; Dimension Icon). The noncontact mode in the air was used to obtain images over a 10 μm–by–10 μm surface area. The 2D height and 3D surface roughness were analyzed using NanoScope Analysis software.

### X-ray photoelectron spectroscopy

XPS (Thermo Scientific K-Alpha) was used to detect the cross-linking degree of the membrane. The spectrometer is equipped with a monochromatic Al Kα X-ray source (hν = 1486.6 eV) operating at voltage 12 kV, filament current 6 mA, under high vacuum (~5.0 × 10^−7^ mbar), using an aperture slot of 5 mm by 5 mm. The full-spectrum scanning pass energy is 150 eV, and the step size is 1 eV. Narrow spectra were collected using a pass energy of 50 eV and a step size of 0.1 eV.

### Fourier transform infrared spectroscopy

FTIR (Bruker TENSOR II) was used to analyze the chemical bond information of the membrane in the spectral range of 400 to 4000 cm^−1^ under the ATR mode.

### Water contact angle

To obtain the hydrophilicity of membranes, the water contact angle was measured using a contact angle instrument (JY-82C), wherein 2 μl of water droplets at three different locations was recorded to get the average data.

### Grazing-incidence wide-angle x-ray scattering

The GIWAXS (Xeuss 2.0) measurements of the nanofilms were performed on a French company Xenocs equipped with a Pilatus 3R 300K detector. Before measurement, the membrane samples were transferred onto a silicon substrate and dried, and all samples were placed under a vacuum to eliminate atmospheric scatter.

### Tensile strength testing of membranes

The tensile properties of the film were measured by an electronic tensile testing machine (Instron 5943, America) with a tensile rate of 10 mm/min controlled by a microcomputer. Before the test, the film sample was cut into 13 mm by 40 mm strips according to the standard method for tensile strength test.

### Young’s modulus measurement

Young’s modulus was measured in PFQNM mode on the Dimension Icon AFM system (AFM, Dimension Icon). Before measurement, the freestanding nanofilm samples were transferred to the silicon substrate and dried at room temperature. The noncontact mode in the air was used to obtain images over a 5 μm–by–5 μm surface area. The measurements were carried out at room temperature and a relative humidity of 30%.

### Brunauer-Emmett-Teller

The Brunauer-Emmett-Teller (Quantachrome Autosorb IQ) technique was used to measure the pore structure of the prepared membrane. The film has been freeze-dried for 10 hours before degassing. Membrane samples were degassed under a high vacuum at 120°C for 8 hours before analysis.

### Molecular dynamics simulation

The chemical structures of monomers were adopted for constructing PAR (PN, TN, PXN, and TMC), while PACKMOL was carried out to generate the amorphous polymer models. In the MD simulations, the motions of the molecules are all described by Newton’s equation and solved using the velocity-Verlet algorithm. The partial charge of molecules was calculated using Gaussian 16 code and the 6-311G(d,p) basis functions were applied. The GAFF force field was used to describe the TMC, PN, PXN, and TN molecules. The molecular force field consists of nonbonded and bonded interactions. The nonbonded interaction contains van der Waals (vdW) and electrostatic interaction, which is described by Eqs. 1 and 2, respectively

$$E_{\mathrm{LJ}}\left(r_{\mathrm{ij}}\right)=4\varepsilon_{\mathrm{ij}}\left({\left(\frac{\sigma_{\mathrm{ij}}}{r_{\mathrm{ij}}}\right)}^{12}-{\left(\frac{\sigma_{\mathrm{ij}}}{r_{\mathrm{ij}}}\right)}^{6}\right)$$ (1)

$$E_{c}\left(r_{\mathrm{ij}}\right)=\frac{q_{i}q_{j}}{4\pi \varepsilon_{0}\varepsilon_{r}r_{\mathrm{ij}}}$$ (2)

$$\sigma_{\mathrm{ij}}=\frac{1}{2}\left(\sigma_{\mathrm{ii}}+\sigma_{\mathrm{jj}}\right);\varepsilon_{\mathrm{ij}}={\left(\varepsilon_{\mathrm{ii}}*\varepsilon_{\mathrm{jj}}\right)}^{\frac{1}{2}}$$ (3)

In the equations, *q_i_* and *q_j_* are atomic charges, *r_ij_* is the distance between atoms, σ is the atomic diameter, and ε is the atomic energy parameter. For different kinds of atoms, the geometric mix rules were adopted for vdW interactions, which follows Eq. 3. The cutoff distance of vdW and electronic interactions was set to 1.2 nm, and the particle-particle-particle mesh (pppm) method was used to calculate long-range electrostatic interactions. For the simulation, an energy minimization was first used to relax the simulation box.

### Organic solvent nanofiltration performance test

OSN experiments were carried out using a homemade dead-end filter device, which was mainly driven by nitrogen at room temperature (effective membrane area of 2.8 cm^2^). The membrane was immersed in the test solvent for a period of time before testing, and after it was stabilized, the feed solution with a solute concentration of 30 ppm in methanol was poured into the cell, and the rotational speed of 500 rpm was maintained during the test to reduce concentration polarization. The solvent permeance (*J*, in liters per square meter per hour per bar) was calculated using Eq. 4

J=V/A×t×∆p (4)

where *V* is the volume of the solvent, *A* is the effective area of the membrane, *t* is the experimental time, and ∆*p* is the penetration pressure difference as the impetus, respectively.

The concentration of solute in the solution is determined by a UV visible spectrophotometer according to the wavelength of solute and solvent. The rejection *R* (%) was calculated using Eq. 5

$$R=\left(1-C_{p}/C_{f}\right)\times 100\%$$ (5)

where *C*_p_ and *C*_f_ are the concentration of permeate and feed, respectively. Unless otherwise specified, the concentration of the feed solution used is fixed at 30 ppm, the pressure is set to 6 bar, and the results are calculated using at least three identical membranes.

## Supplementary Materials

### This PDF file includes:

Figs. S1 to S39 Tables S1 to S6 References
