# Supplementary material for: Microporous polyarylate membranes based on 3D phenolphthalein for molecular sieving
Source: Sci Adv. 2024 Aug 9;10(32):eado7687. doi: 10.1126/sciadv.ado7687 (PMC11313862; doi:10.1126/sciadv.ado7687)
Supplement: Supplementary file 1 — Figs. S1 to S39 Tables S1 to S6 References [file sciadv.ado7687_sm.pdf]

Science Advances
AAAS

Supplementary Materials for

Microporous polyarylate membranes based on 3D phenolphthalein for molecular sieving

Ayan Yao *et al.*

Corresponding author: Jiangtao Liu, jiangtaoliu@ustc.edu.cn

Sci. Adv. **10**, eado7687 (2024)
DOI: 10.1126/sciadv.ado7687

The PDF file includes:

Figs. S1 to S39
Tables S1 to S6
References

Other Supplementary Material for this manuscript includes the following:

Movies S1 to S3

Supplementary figure and table

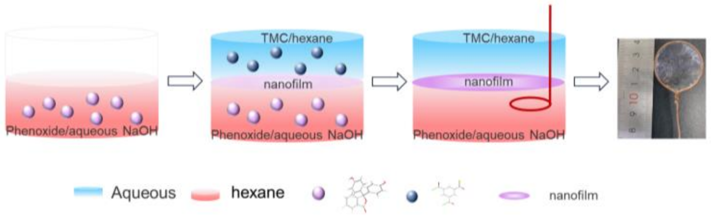

Fig. S1 Schematic presentation of the fabrication process of freestanding TMC-PN nanofilm. PN and TMC monomer were first dissolved in aqueous NaOH solution and n-hexane, respectively. Subsequently, the resulting free-standing nanofilm was scooped out the interface with a wire loop and was rinsed several times by the deionized water and hexane to wash away the residual monomers.

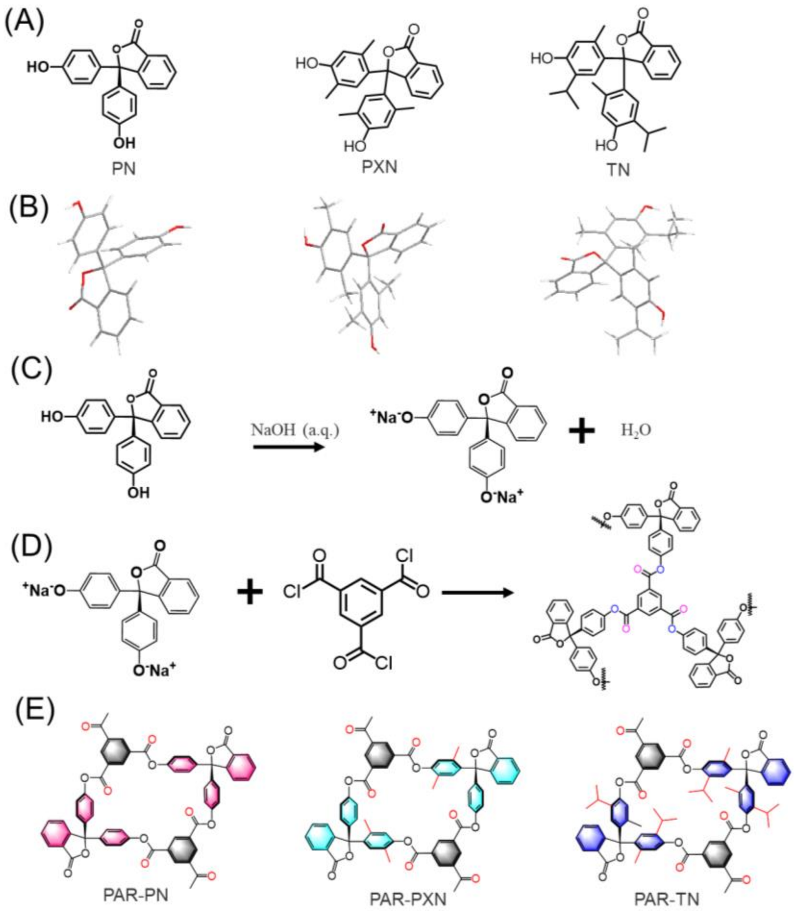

Fig. S2 Synthesis of polyarylate network polymers by interfacial polymerisation. (A) Three different phenol monomers were used to form four crosslinked polyarylate materials: including phenolphthalein (PN), P-Xylenolphthalein (PXN) and Thymolphthalein (TN); (B) Molecular model of three phenol monomers used. Each phenol was dissolved in a dilute sodium hydroxide aqueous solution and reacted interfacially with trimesoyl chloride dissolved in hexane; (C, formation of PN phenoxide when phenol is dissolved in diluted NaOH aqueous solution; (D, shows the reaction of PN phenoxide with trimesoyl chloride to form a polyarylate network; (E) Proposed pore structure of PAR membranes.

Table S1 Structural parameters derived from experimental and simulation data. Experimentally measured density of polyarylates prepared through interfacial polymerization reaction versus simulated density values.

Membrane type		Occupied volume (Å³)	Free Volume (Å³)	Porosity (%)	Density (g/cm³)	Largest Pore size (Å)
PAR-PN	experimental	-	-	17	1.26	8.7
	simulation	258344.11	68769.59	21	0.83	8
PAR-PXN	experimental	-	-	17	1.16	8.9
	simulation	290916.10	98970.45	25.3	0.79	11
PAR-TN	experimental	-	-	23	1.08	9.1
	simulation	323971.24	127707.26	28.3	0.75	12

Note: Classic molecular dynamics simulations were carried out to investigate the mixed solution from the atomic level. Three cases (PAR-PN, PAR-PXN, PAR-TN) were built for molecular dynamic simulations. Case PAR-PN contains 400 TMC, 600 PN molecules. Case PAR-PXN contains 400 TMC, 600 PXN molecules. Case PAR-TN contains 400 TMC, 600 TN molecules. The initial configurations systems were constructed through the software of PACKMOL, all the molecules were randomly inserted in a cubic simulation box. Cell size: $70 \times 70 \times 70$ Å^3. In the simulation, the phenolic monomer and TMC are freely cross-linked at room temperature and atmospheric pressure until the number of new cross-linked products no longer increases, and the size of the simulation box basically remains unchanged. At this time, the simulation system has been stabilized and the structural parameters of each case are recorded.

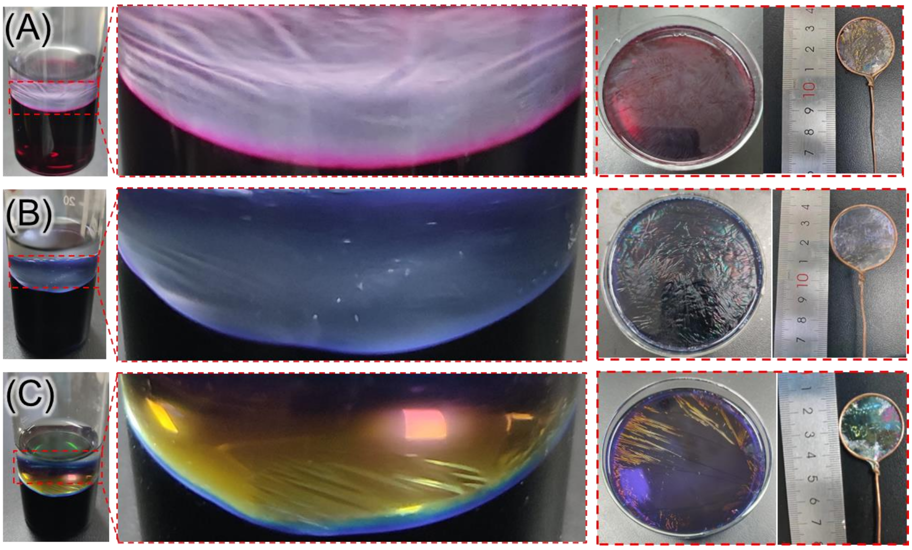

Fig. S3 Polyarylate nanofilms. Photographs of the free-standing polyarylate nanofilm formed at the free organic/water interface, floating in liquid surface and captured by a wire loop. (A) PAR-PN, (B) PAR-PXN, (C) PAR-TN.

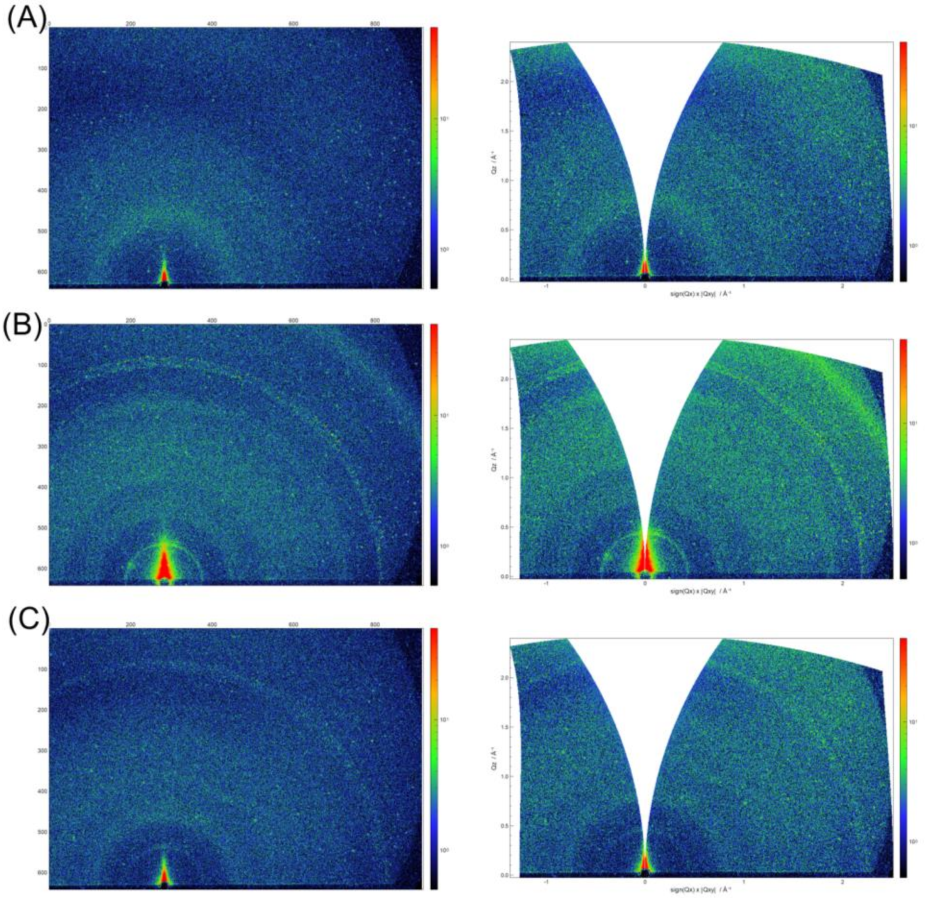

Fig. S4 . Characterizations of PAR membranes. Grazing incidence wide-angle X-ray scattering (GIWAXS) two-dimensional images of PAR nanofilms on a SiO_2/Si substrate. (A) PA-PN, (B) PAR-PXN and (C) PAR-TN.

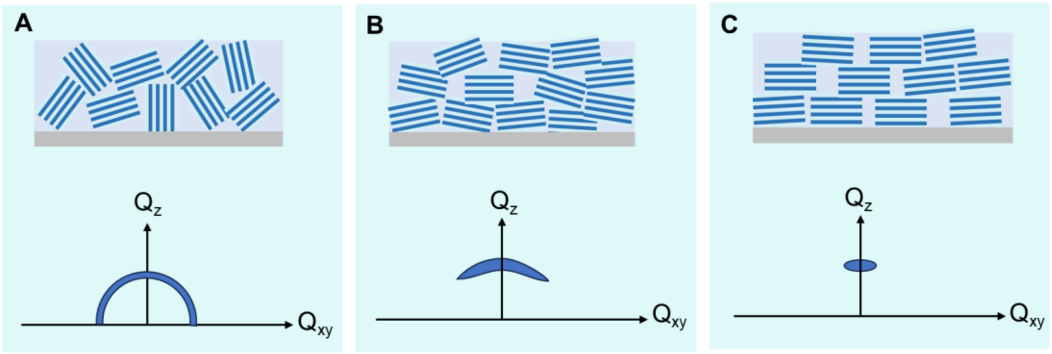

Fig. S5 Possible texture in thin films. (A) Randomly oriented arrangements of crystallites, with no preference for a specific crystallographic orientation with respect to the substrate normal produce rings in the diffraction patterns. (B) Textured or oriented films with a distribution of crystallite orientations produce arcs of diffracted intensity. (C) Highly oriented films produce spots or ellipses. Note: the shape and intensity distribution of diffraction peaks in reciprocal (Qxy-Qz) space can be used to describe the orientation of the diffracting crystallites. Nanofilms without preferred crystallographic orientation will result in a ring of uniform intensity.

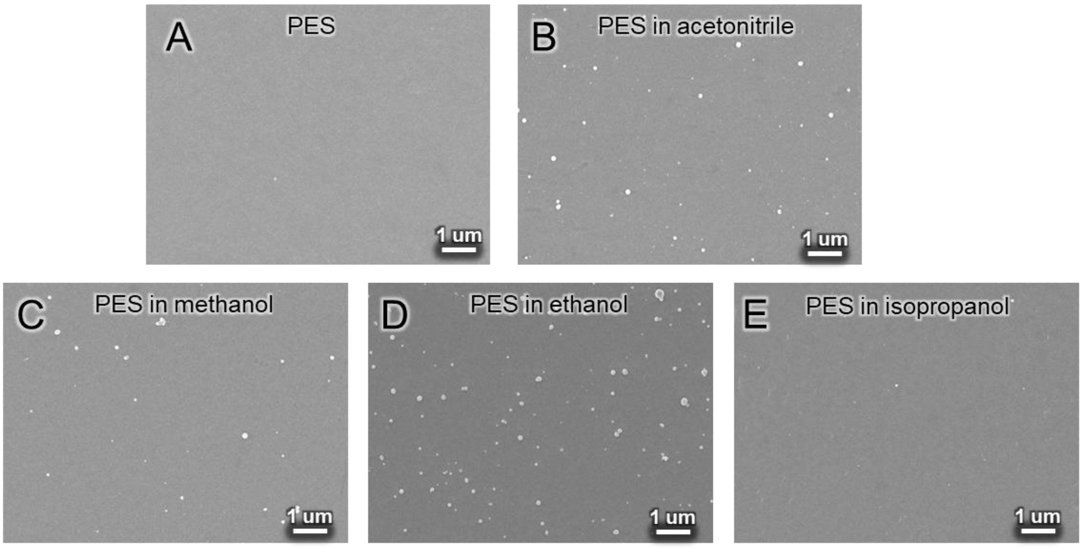

Fig. S6 Characterizations of PAR membranes. SEM images of PES substrate immersed in various organic solvents after 7 days. (A) PES substrate, (B) PES in acetonitrile, (C) PES in methanol, (D) PES in ethanol, (E) PES in isopropanol.

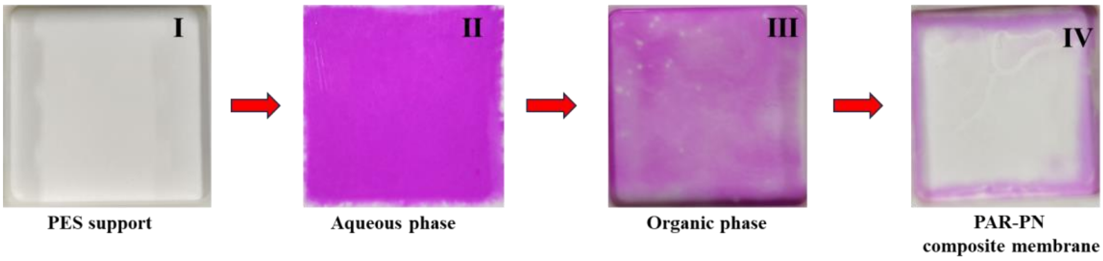

Fig. S7 The preparation process of PAR-PN TFC membrane by interfacial polymerisation. Note: (I) represents PES substrate, (II) represents the surface of the membrane after immersion in PN solution of sodium hydroxide, (III) represents the surface of the membrane during the reaction, and (IV) represents the surface of the prepared TFC membrane.

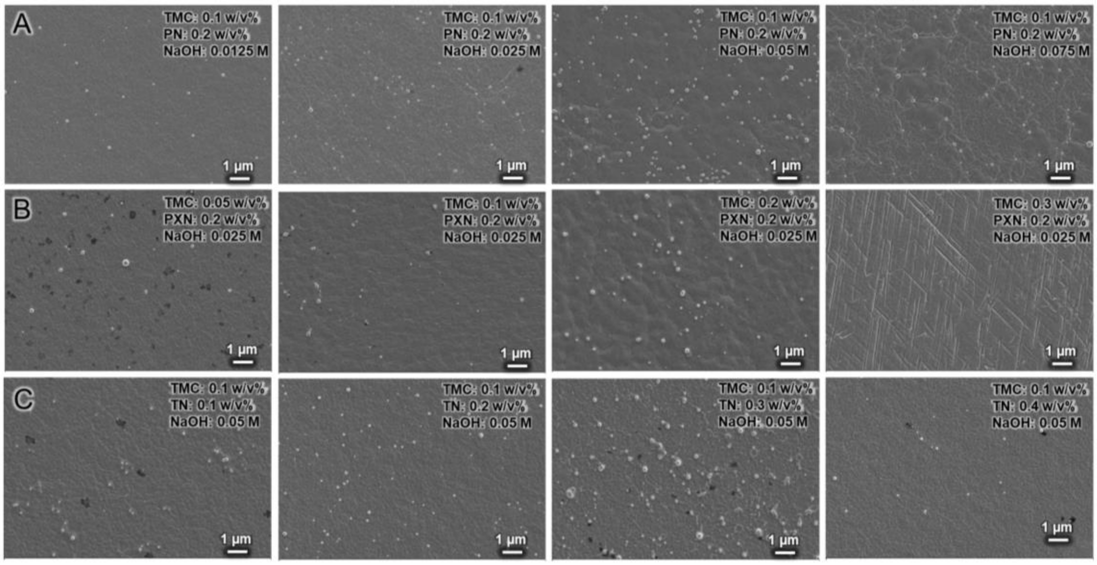

Fig. S8 Characterizations of PAR membranes. Representative SEM images of the top surface of polyarylate membranes at different conditions. (A) NaOH, (B) TMC, and (C) TN.

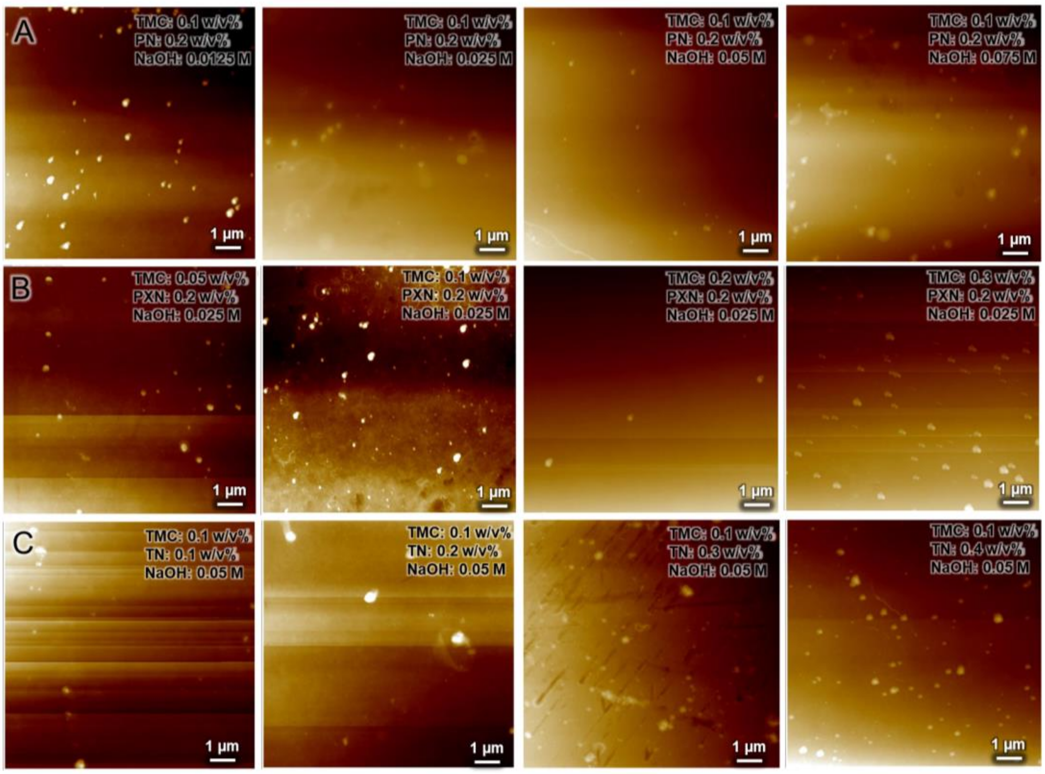

Fig. S9 Characterizations of PAR membranes. 2D AFM images of the top surface of polyarylate membranes at different conditions. (A) NaOH, (B) TMC, and (C) TN.

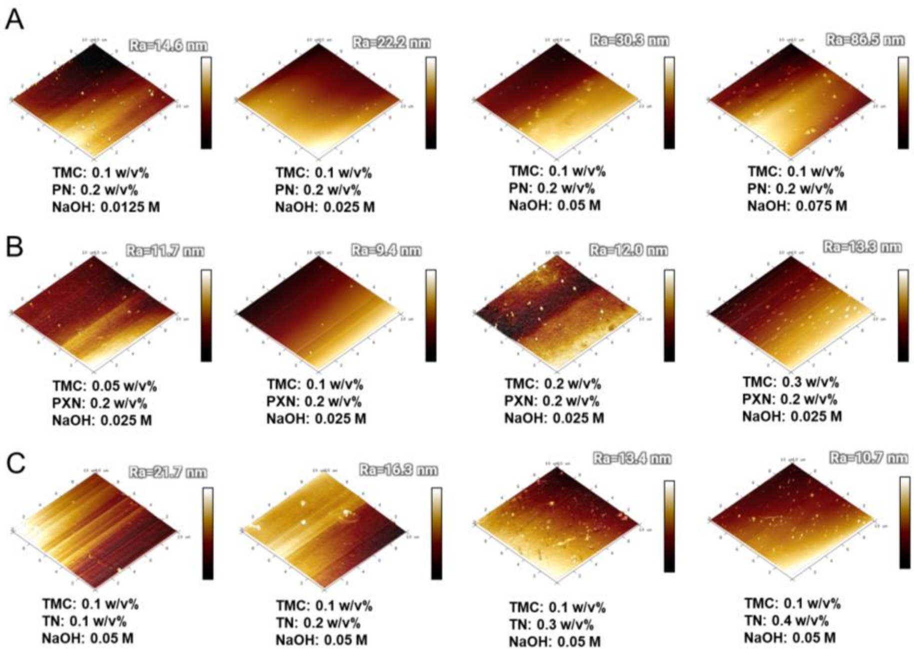

Fig. S10 Characterizations of PAR membranes. 3D AFM images of the top surface of polyarylate membranes at different conditions. (A) NaOH, (B) TMC, and (C) TN.

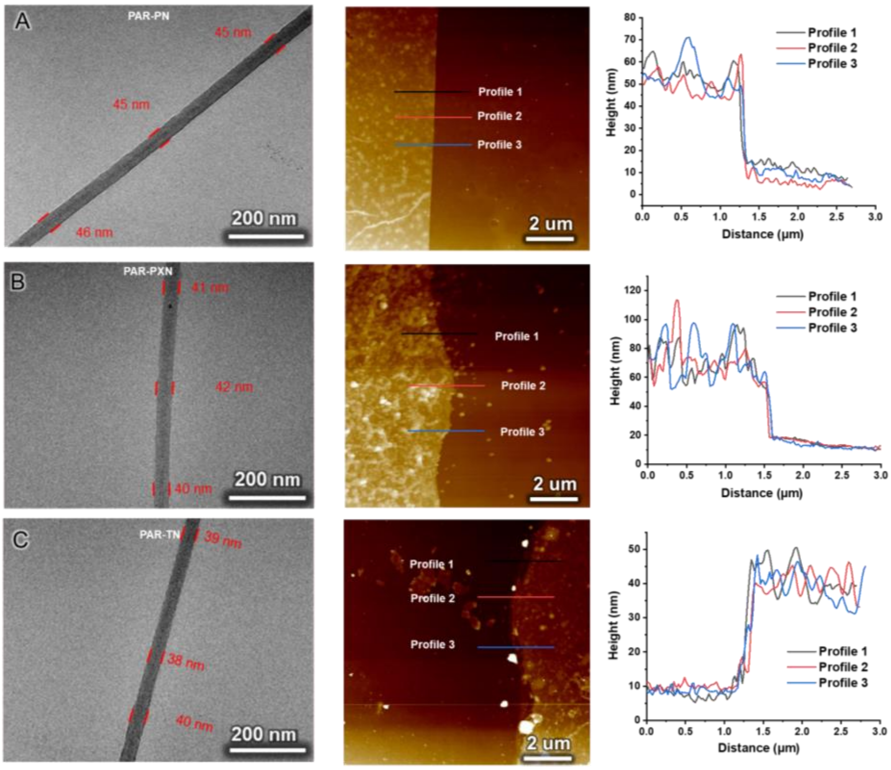

Fig. S11 Characterizations of PAR membranes. TEM cross-sectional images, AFM image and corresponding height profile of a section of a PAR nanofilm on top of a silicon wafer. A scratch was made to expose the wafer surface and allow measurement of the height from the silicon wafer surface to the upper nanofilm surface. (A) PAR-PN, (B) PAR-PXN, and (C) PAR-TN.

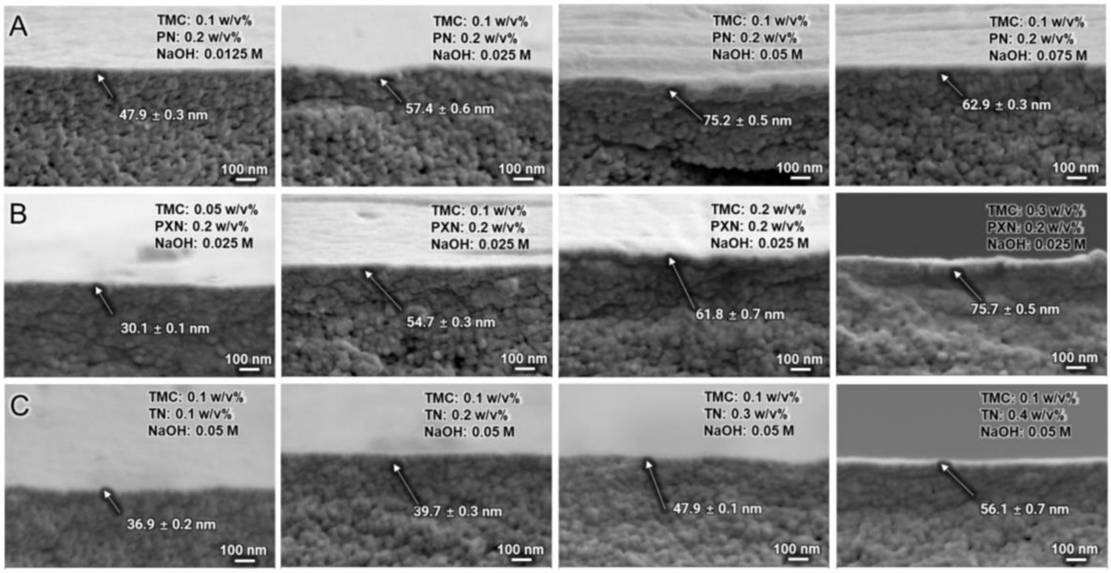

Fig. S12 Characterizations of PAR membranes. SEM images of the cross section of polyarylate membranes at different conditions. (A) NaOH, (B) TMC, and (C) TN.

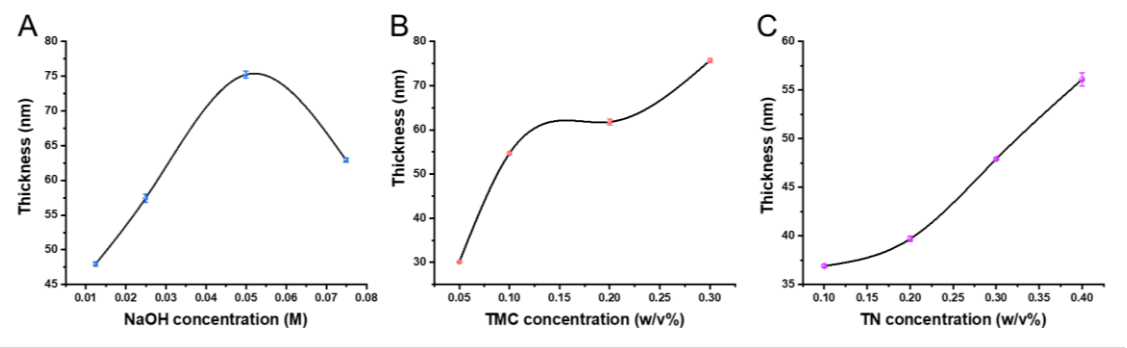

Fig. S13 Thickness characterizations of PAR membranes. Thickness of polyarylate TFC membranes at different concentrations of (A) NaOH, (B) TMC and (C) TN. Note: as analyzed by SEM.

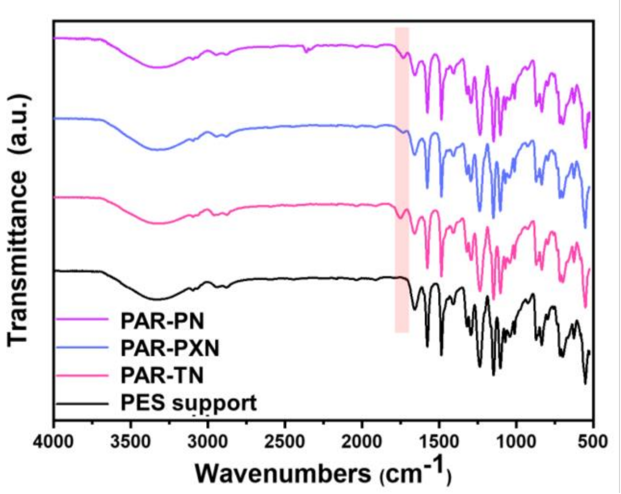

Fig. S14 FTIR spectra of polyarylate nanofilms and PES support membrane over 4000-500 cm^{-1}. The FTIR spectra show the carbonyl group (C=O) stretching at about 1740-1730 cm^{-1}, which corresponds to the C=O stretching in the ester groups.

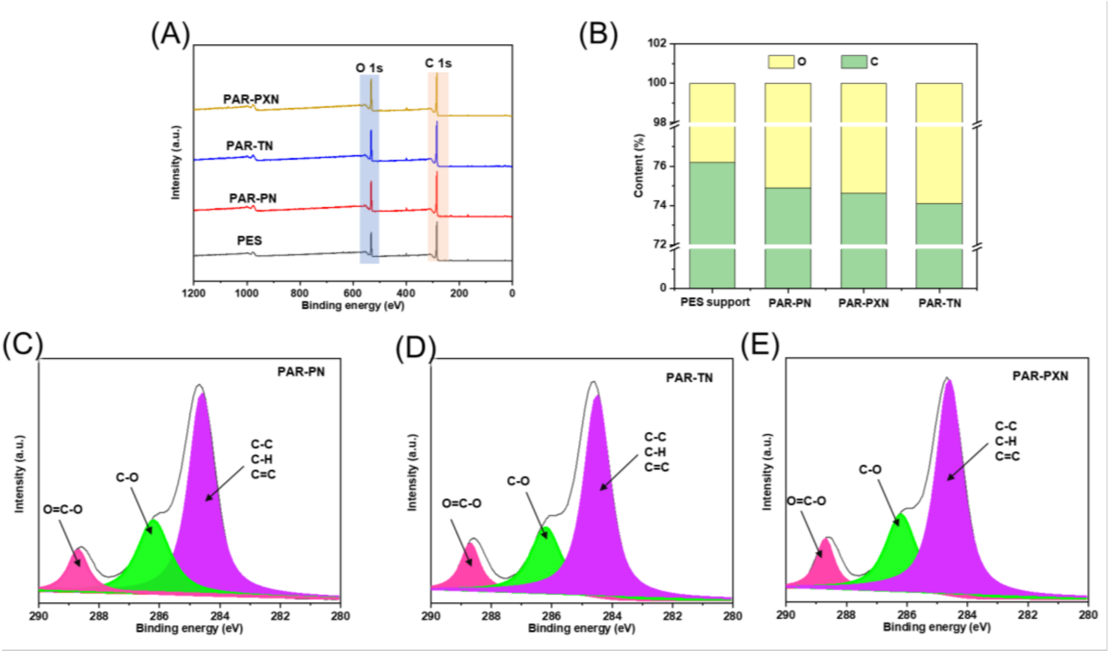

Fig. S15 High-resolution XPS spectra of PAR membranes. (A) XPS spectra of the PES support membrane and polyarylate nanofilms. (B) XPS results of polyester membranes. (C, D and E) High-resolution XPS spectra of C 1s of polyarylate nanofilms.

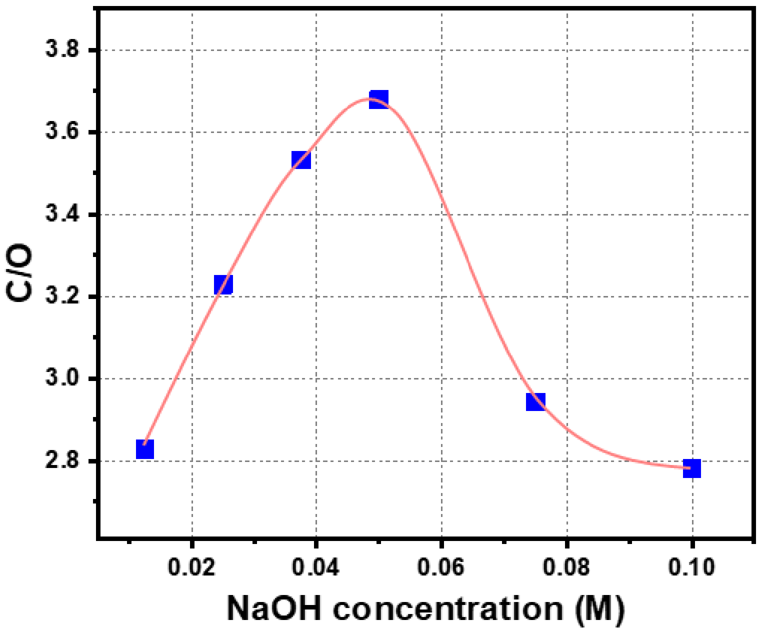

Fig. S16 Crosslinking degree of the nanofilm with different NaOH concentrations. Note: as analyzed by C/O ratio measured by XPS.

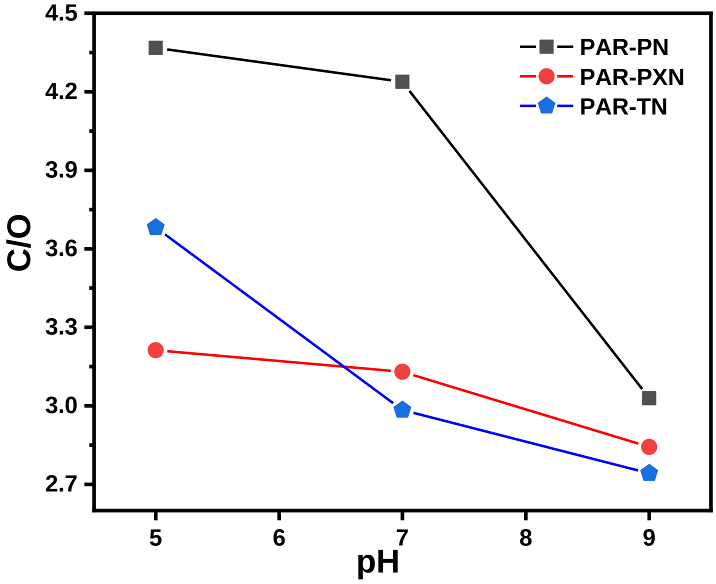

Fig. S17 C/O ratio of the membranes with different pH. Note: The exact cross-linking degree of PAR membranes could not be calculated, but it can be roughly compared according to content of C/O ratio. The prepared three membranes were soaked in feed solution at different pH for 6 h. As analyzed by C/O ratio measured by XPS.

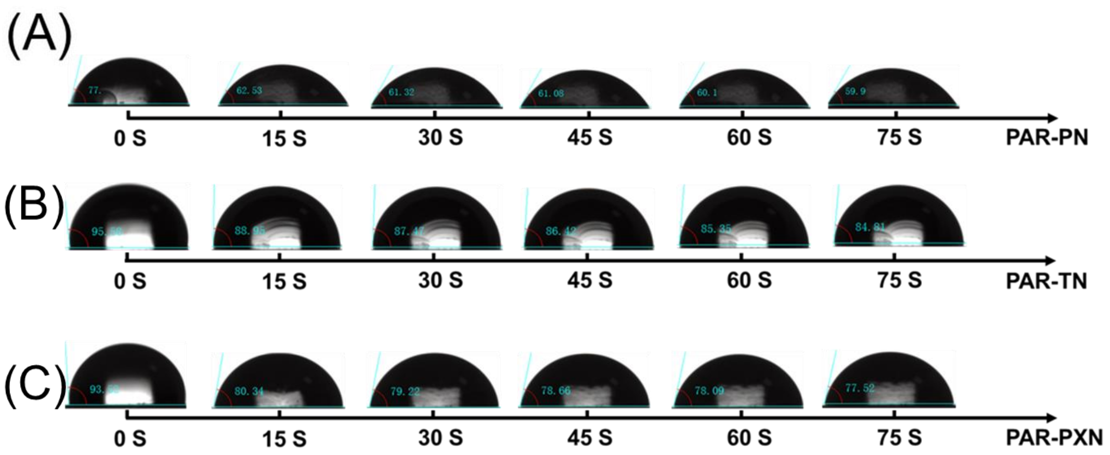

Fig. S18 Water contact angle of polyarylate membranes. Measurement of water contact angle over wetting time of 75 sec. (A) PA-PN, (B) PAR-TN and (C) PAR-PXN membranes.

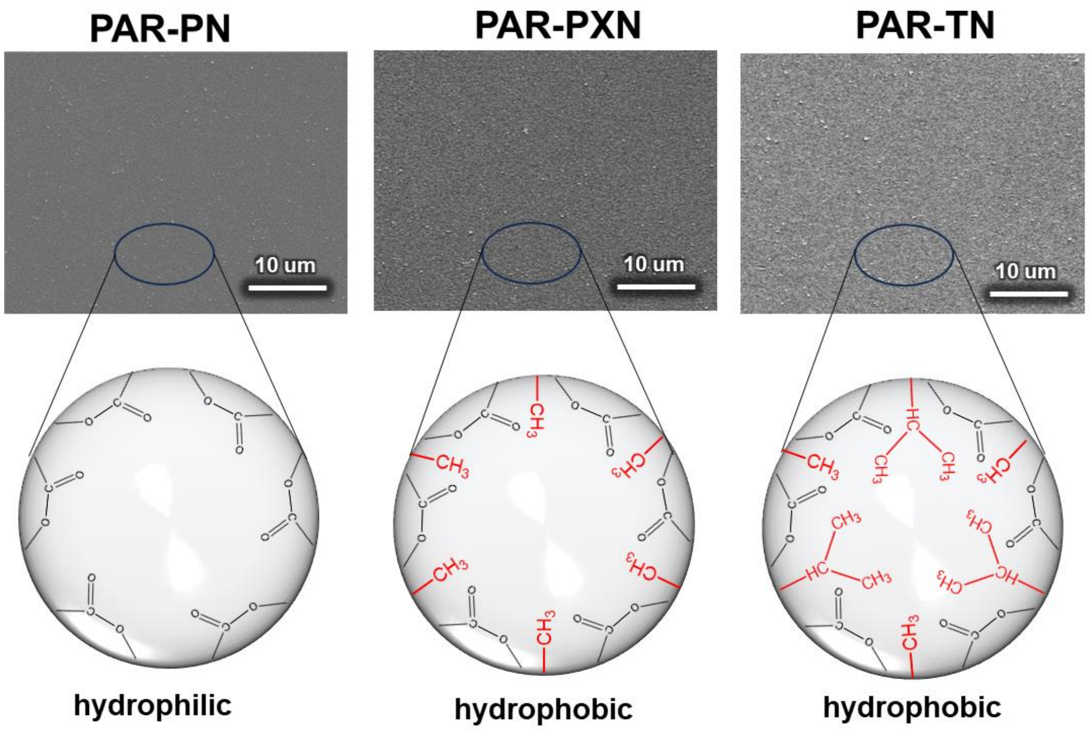

Fig. S19 Surface chemical structure model of PAR membrane. SEM image and chemical composition diagram of the surfaces of PAR-PN,PAR-PXN and PAR-TN membranes.

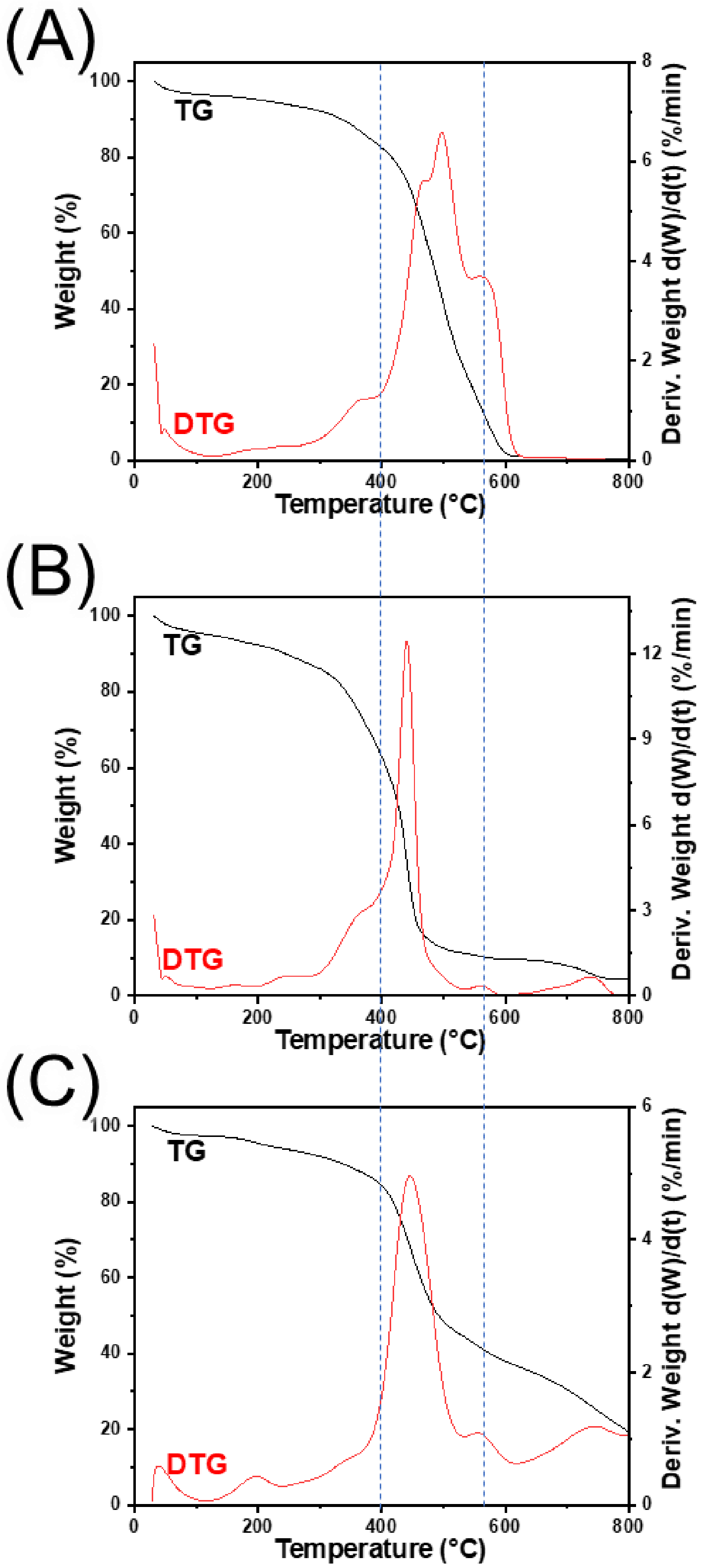

Fig. S20 Thermal stability characteristics of PAR membranes. TGA curves of polymer powders synthesized via interfacial polymerisation obtained by rigorous mixing of a solution of TMC in hexane added to a solution of each phenol in NaOH/water. (A) PAR-PN, (B) PAR-PXN, and (C) PAR-TN.

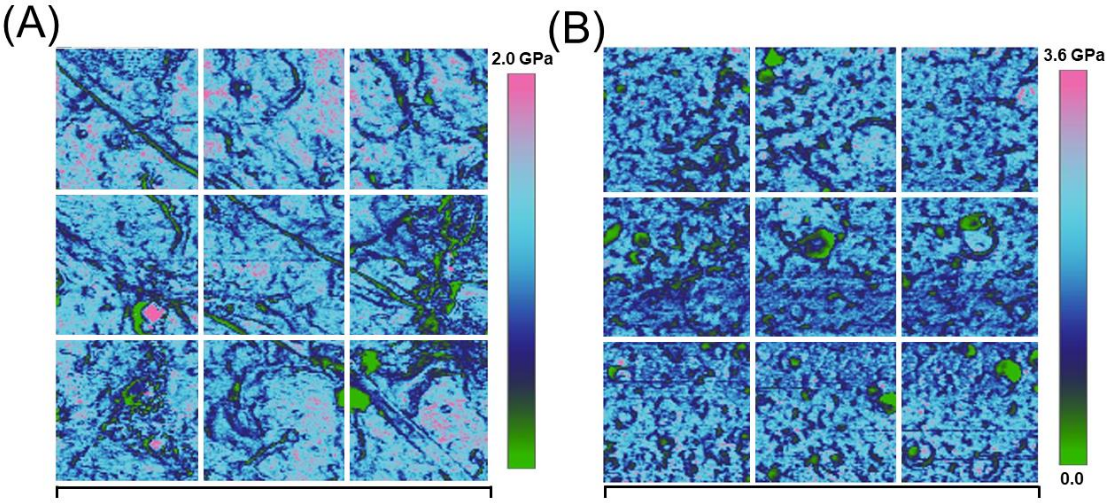

Fig. S21 Mechanical strength. Young's moduli of polyarylate nanofilms and polyamide nanofilm, tested using the PFQNM method. (A) PAR-PN, (B) PAR-PXN membrane. Scale bar: 5 μm.

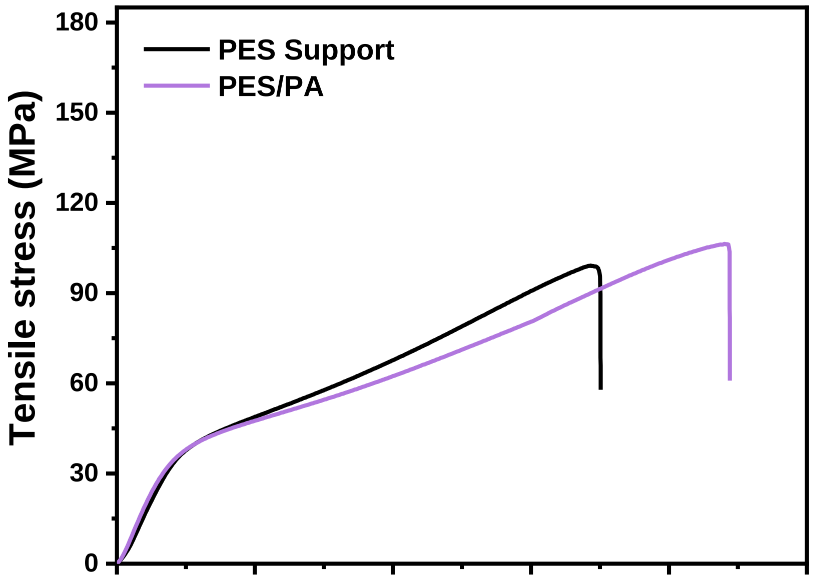

Fig. S22 Mechanical properties testing. Stress-strain curves of the PES support and PA composite membranes by interfacial polymerization, tested using a microcomputercontrolled electronic tensile testing machine.

Table S2 Chemical composition, water contact angle, and Young's modulus of polyarylate nanofilms. Chemical composition is based on atomic percentage.

Nanofilm	Chemical composition (%)		Contact angle	Young's modulus (GPa)	tensile strength (MPa)	Elongation (%)
	Carbon	Oxygen				
PES	76.19	23.81	-	-	99.1	17
PAR-TN	74.11	25.89	95.7±0.2°	2.4	163.3	19.9
PAR-PXN	74.63	25.37	93.4±0.2°	1.6	155.7	17.1
PAR-PN	74.9	25.1	77.1±0.1°	1.0	105.1	21
PA	-	-	-	0.8	106.3	22

Table S3. Physical properties of solvents used for nanofiltration tests.

Solvent	Molecular weight ($g\ mol^{-1}$)	Kinetic diameter (nm)	Viscosity (η) at 25 °C (mPa·S)	Solubility Parameter δ ($MPa^{1/2}$)
methanol	32.0	0.51	0.49	12.3
ethanol	46.1	0.57	1.17	8.8
acetone	58.1	0.62	0.29	10.4
isopropanol	60.1	0.62	2.1	6.1

δ= solubility parameter due to dipole forces.

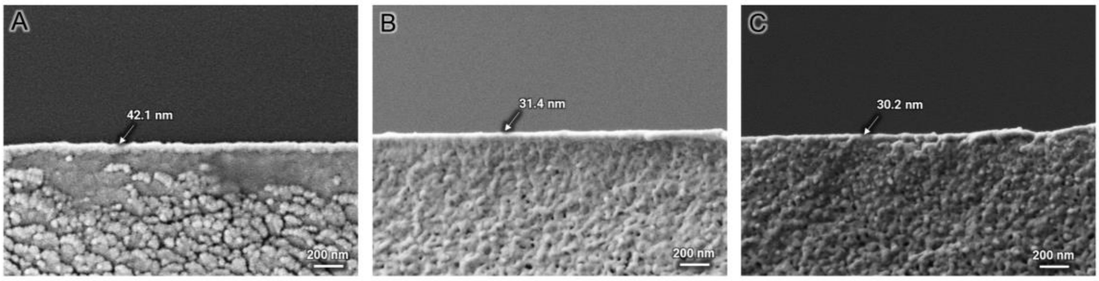

Fig. S23 SEM images of the cross section of polyarylate TFC membranes. (A) PAR-PN, (B) PAR-PXN, and (C) PAR-TN membrane. The mmembrane was prepared by interfacial polymerisation of TMC (0.1 w/v%) in hexane with phenols (0.2 w/v%) in NaOH/water on PES supports.

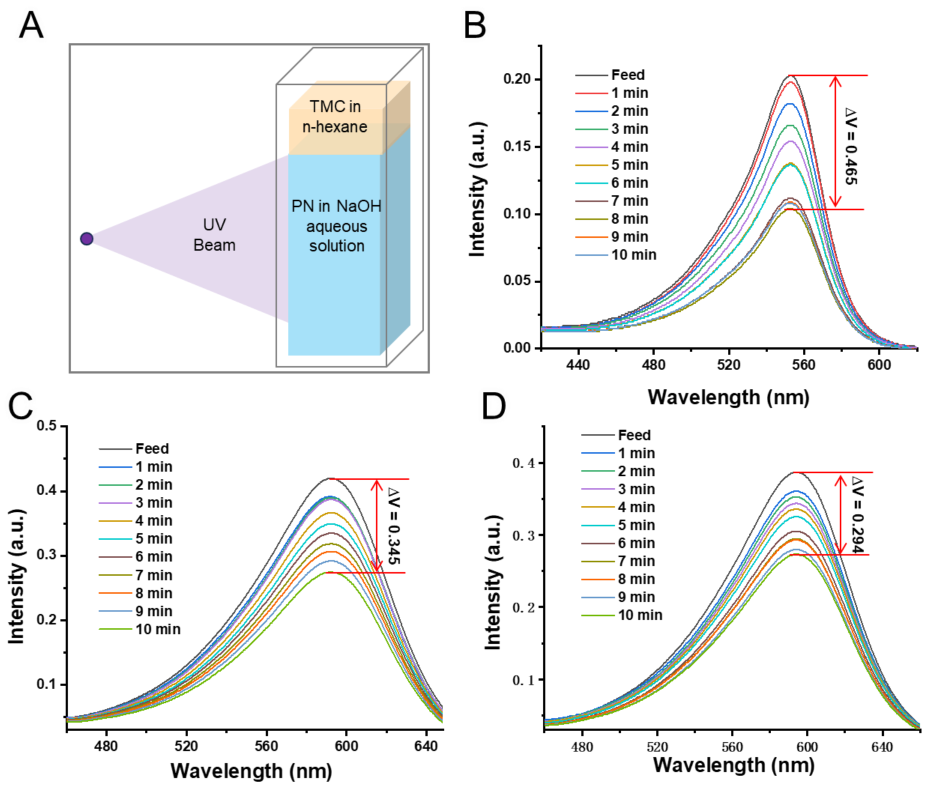

Fig. S24 Diffusion experiment. (A) Schematic illustration of in situ monitoring of interfacial diffusion by UV-Vis spectrophotometry in a fixed quartz cuvette. Time-dependent UV-vis spectra changes of monomers in aqueous phase during IP process, (B) PN, (C) PXN, (D) TN. Note: Compared with PXN and TN, the diffusion rate of monomers from water to n-hexane phase follows the order of PN > PXN > TN.

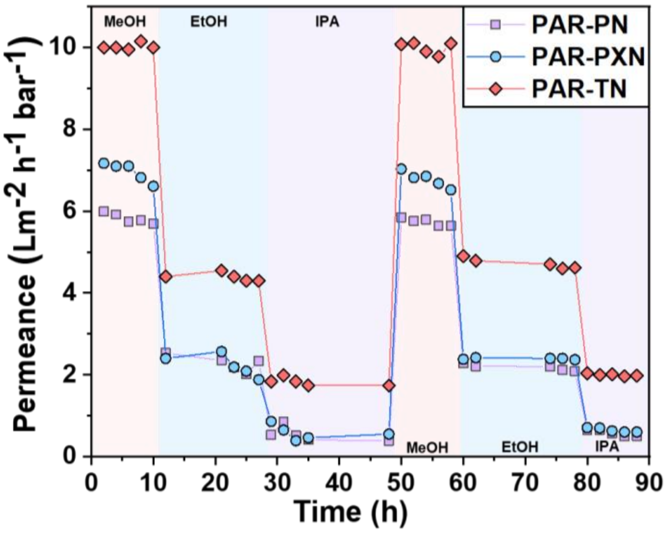

Fig. S25. Solvent stability test. Plot of isopropanol, methanol and ethanol permeances with time for PAR membranes.

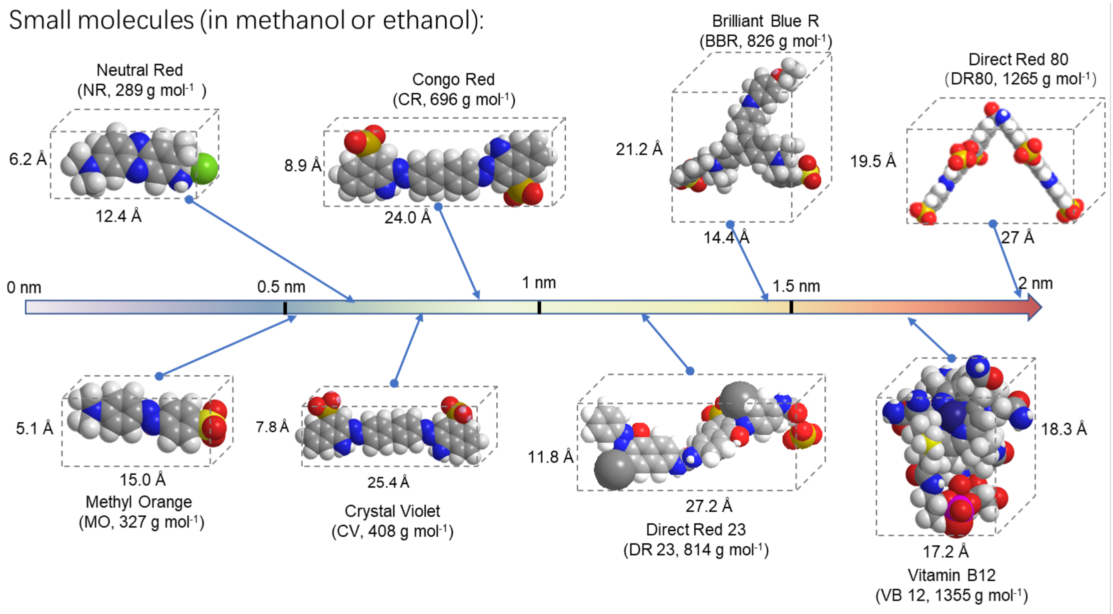

Fig. S26 Schematic of spacing filling models for solutes involved in the solute separation experiments with arrows linking to the corresponding solute diameter. Note: the geometric mean diameters are derived from the molecular dimensions estimated by the Chem3D software package for dye molecules.

Table S4. Organic solvent nanofiltration performance of nanofilm composite membranes. Dyes with varying molecular weight dissolved in methanol were used to study solute retention. Nanofiltration experiments were conducted in a dead-end stirred cell at 25°C under 7 bar.

Membrane (PAR/TMC-w/v%-phenols-w/v)	MeOH permeance ($Lm^{-2}h^{-1}bar^{-1}$)	Dye rejection (%)								NaOH (w/v%)
		MO	NR	CV	CR	DR23	BBR	VB12	DR80	
		(0.51nm)	(0.62nm)	(0.78nm)	(0.89nm)	(1.18nm)	(1.44nm)	(1.72nm)	(1.95nm)	
PAR/TMC-0.05-PN-0.2	7.1±0.2	2.5±0.9	43.1±3.6	59.6±3.3	77.5±0.5	91.8±1.4	94.3±0.3	94.5±0.5	99.4±0.2	0.1
PAR/TMC-0.1-PN-0.2	4.9±0.1	58.3±10.3	74.9±5.7	78.6±0.8	92.4±0.7	98.1±0.7	95.4±0.2	94±0.5	97.8±0.6	0.1
PAR/TMC-0.1-PN-0.2	5.4±0.1	35.6±0.6	64.9±2.4	66±7.7	86.7±2.4	93.2±4.0	93.0±0.1	92±0.1	98.8±0.1	0.2
PAR/TMC-0.1-PN-0.2	9.9±0.1	26.5±2.3	57.7±0.1	63.4±0.1	77.6±0.2	93.2±4.1	93±0.1	92±0.5	99.8±0.1	0.3
PAR/TMC-0.1-PN-0.3	7.2±0.1	56.9±3.2	90.1±0.7	81.4±0.2	93.7±1.9	98.1±0.7	95±0.1	93.4±0.5	98.4±0.1	0.2
PAR/TMC-0.1-PN-0.4	7.3±0.1	60.1±0.9	80±2.3	64.8±0.9	95.4±1.2	92.9±1.9	96.1±0.1	97.1±0.2	97.6±0.1	0.2
PAR/TMC-0.05-PXN-0.2	5.9±0.1	22.4±6.3	49±1.0	74.3±1.2	63.5±2.4	91.5±0.1	94.8±0.2	96.8±0.2	96.5±0.1	0.1
PAR/TMC-0.1-PXN-0.2	5.5±0.2	28.5±3.3	64.6±3.8	58.9±4.4	93.5±0.3	98.2±0.3	93.1±0.9	96.4±0.5	98±0.1	0.1
PAR/TMC-0.2-PXN-0.2	5.8±0.9	36.9±11.4	81.2±0.5	69.2±2.2	96.7±0.4	93±1.8	90.7±0.9	91.3±0.5	97.9±0.4	0.1
PAR/TMC-0.3-PXN-0.2	3.9±0.3	86.2±1.4	94.4±0.5	90.3±0.5	96±0.1	97.1±0.3	92±0.2	98.3±1.5	98.9±0.1	0.1
PAR/TMC-0.05-TN-0.2	7.4±0.1	4.8±1.7	20.4±0.2	48.4±7.4	65.8±6.9	92.5±0.1	89.8±3.0	97.6±0.1	97.0±0.3	0.1
PAR/TMC-0.1-TN-0.1	3.6±0.4	40.5±0.5	68.8±0.2	70.4±1.6	95.5±0.2	97±0.1	95.5±0.3	95.8±0.9	97.8±0.2	0.15
PAR/TMC-0.1-TN-0.2	6.3±0.2	23±4.7	78.3±1.5	57.8±2.3	94.8±0.9	93.1±1.8	96.6±0.6	93.3±0.1	97.9±0.3	0.15
PAR/TMC-0.1-TN-0.3	4.1±0.2	54.4±0.8	72±1.2	74±0.1	95.2±0.9	93.2±2.0	97.9±0.2	95.1±1.5	98.2±0.3	0.15
PAR/TMC-0.2-TN-0.2	3.3±0.5	89.6±0.7	91.3±0.4	94.3±1.0	98.6±0.3	97.6±0.7	99.6±0.1	99.6±0.2	89.6±0.7	0.2
PAR/TMC-0.05-PN-0.2	7.1±0.2	2.5±0.9	43.1±3.6	59.6±3.3	77.5±0.5	91.8±1.4	94.3±0.3	94.5±0.5	99.4±0.2	0.1

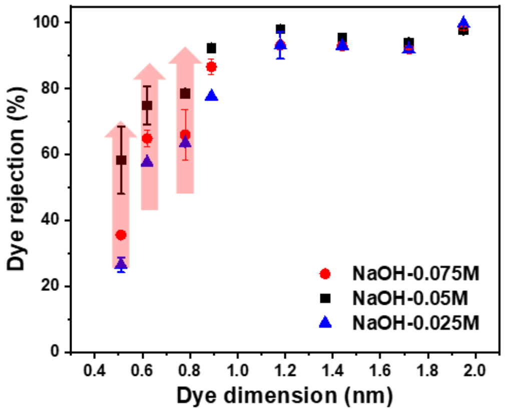

Fig. S27 OSN performance of PAR-PN membranes. Effect of membranes with different NaOH concentrations on rejection performance.

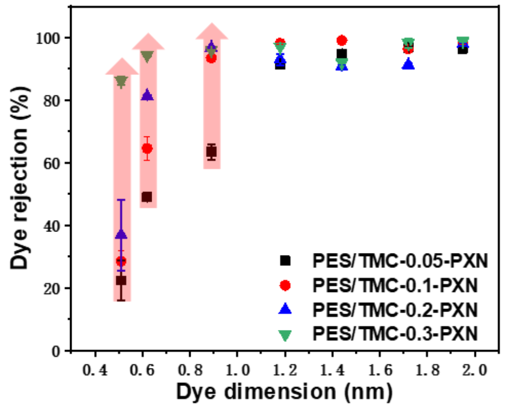

Fig. S28 OSN performance of PAR-PXN membranes. Effect of membranes with different TMC concentrations on rejection.

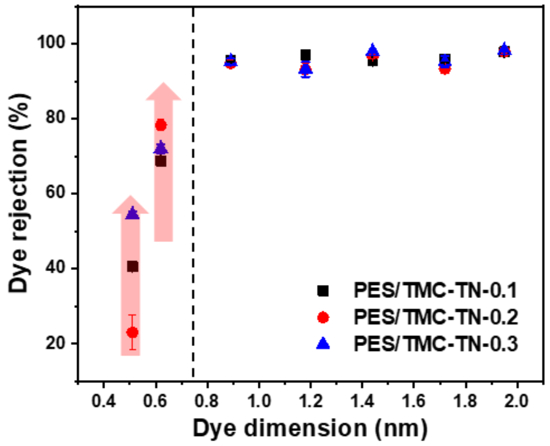

Fig. S29 OSN performance of PAR-TN membranes. Effect of membranes with different TN concentrations on rejection.

Table S5. Summary of solvent permeance of Polyarylate membranes with stateof-the-art polymer membranes.

Support	Membranes	Name	Thickness (nm)	Morphology	MeOH Permeance (L m^{-2} h^{-1} bar^{-1})	
PES	Polyarylate	PAR/TMC-0.05-PN-0.2	61	smooth	7.1	This work
		PAR/TMC-0.05-TN-0.2	43	smooth	7.4	This work
		PAR/TMC-0.05-PXN-0.2	60	smooth	5.9	This work
		PAR/TMC-0.1-PN-0.2	75	smooth	9.9	This work
		PAR/TMC-0.1-PXN-0.2	62	smooth	5.8	This work
		PAR/TMC-0.2-PXN-0.2	55	smooth	5.5	This work
XP84		PAR-BHF	20	smooth	8.0	*(15)*
		PAR-TTSBI	20	smooth	6.0	*(15)*
		PAR-DHAQ	20	smooth	0.6	*(15)*
		PAR-RES	20	smooth	0.6	*(15)*
Alumina	Free-standing polyarylate	PAR-BHPF	200	smooth	4.4	*(15)*
	Free-standing using amino-functionalised macrocycles and acyl chloride	α-CDA-TPC-0.05	10	smooth	7.6	*(45)*
		α-CDA-TPC-0.1	14	smooth	5.8	*(45)*
		β-CDA-TPC-0.1	10.4	smooth	6.3	*(45)*
		β-CDA-TMC-0.1	11	smooth	3.0	*(45)*
		γ-CDA-TPC-0.05	6.2	smooth	9.9	*(45)*
		γ-CDA-TPC-0.1	8.0	smooth	9.3	*(45)*
		γ-CDA-TPC-0.2	13.1	smooth	1.9	*(45)*
		γ-CDA-TPC-2	20.0	smooth	1.6	*(45)*
		SC[4]AA-TPC-0.1	24.9	smooth	3.1	*(45)*

		SC[4]AA-TMC-0.1	21.8	smooth	2.5	*(45)*
Alumina	-	MPD-10%-1min	64	smooth	2.45	*(52)*
Alumina	-	MPD-10%-1min-ACT	47	smooth	6.6	*(52)*
Alumina	-	MPD-0.1%-10min	8.4	smooth	3.14	*(52)*
Alumina	-	MPD-0.1%-10min-ACT	8.0	smooth	12.21	*(52)*
XP84	-	MPD-0.1%-10min	8.4	smooth	3.89	*(52)*
XP84	-	MPD-0.1%-10min-ACT	8.0	smooth	9.55	*(52)*
PAN	-	MPDTrip-20	339	crumpled	8.7	*(13)*
Alumina	-	hydroxyl-BIPOL	14	smooth	13	*(14)*

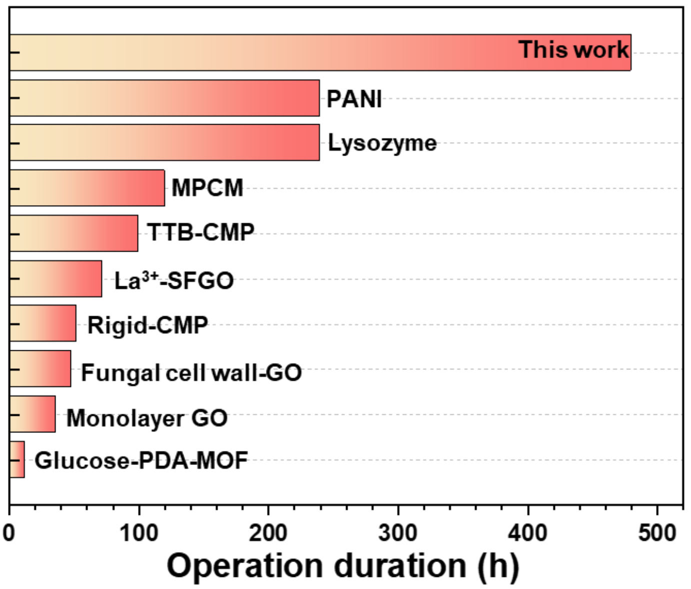

Fig. S30 Comparison of the operational duration between our membrane and others. These data are taken from references *(30, 53-60)*.

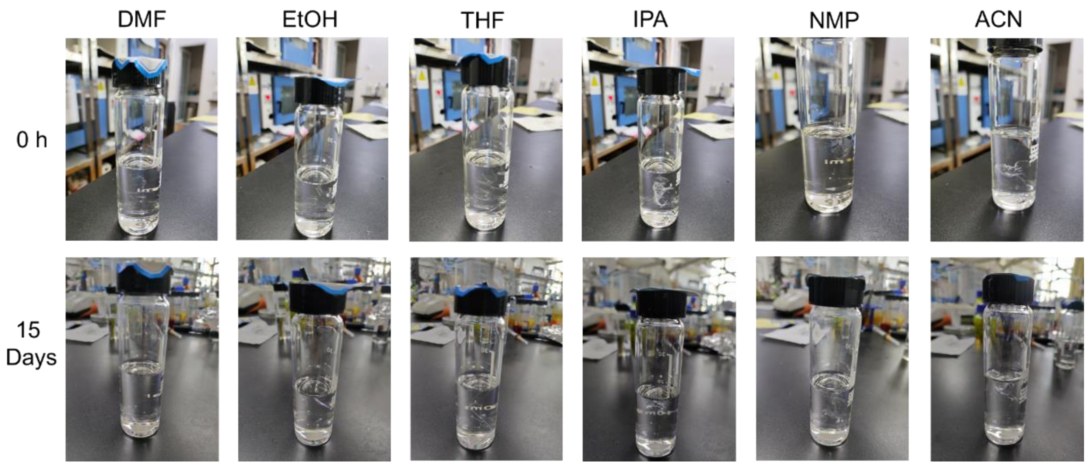

Fig. S31. Images of PAR-TN nanofilms immersed in various organic solvents after 15 days. Note: The polyarylate network has a good tolerance to various organic solvents as demonstrated by their unchanged state after 15 days immersion in various solvents.

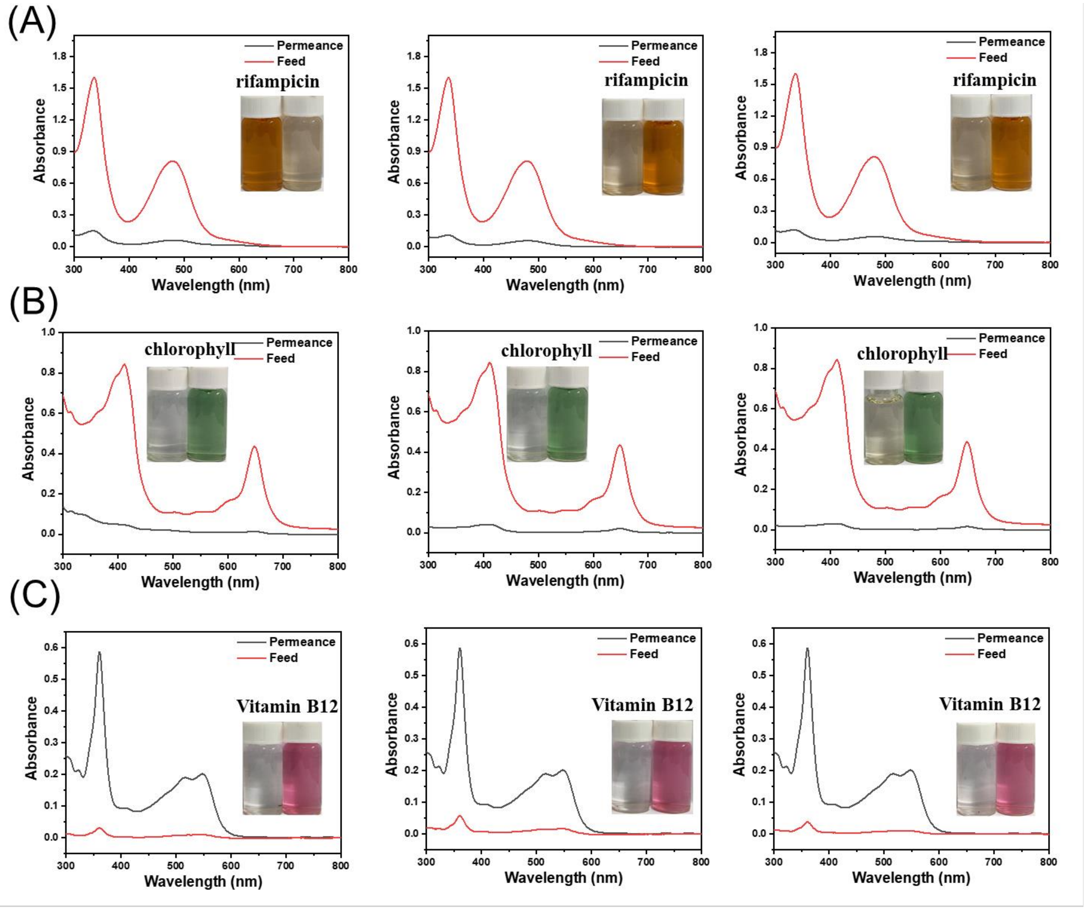

Fig. S32 UV absorption spectra of drugs in ethanol before and after selectivity tests performed with polyarylate membranes. (A) PAR-PN, (B) PAR-PXN, and (C) PAR-TN. Inserts show photographs of the feed and the permeate.

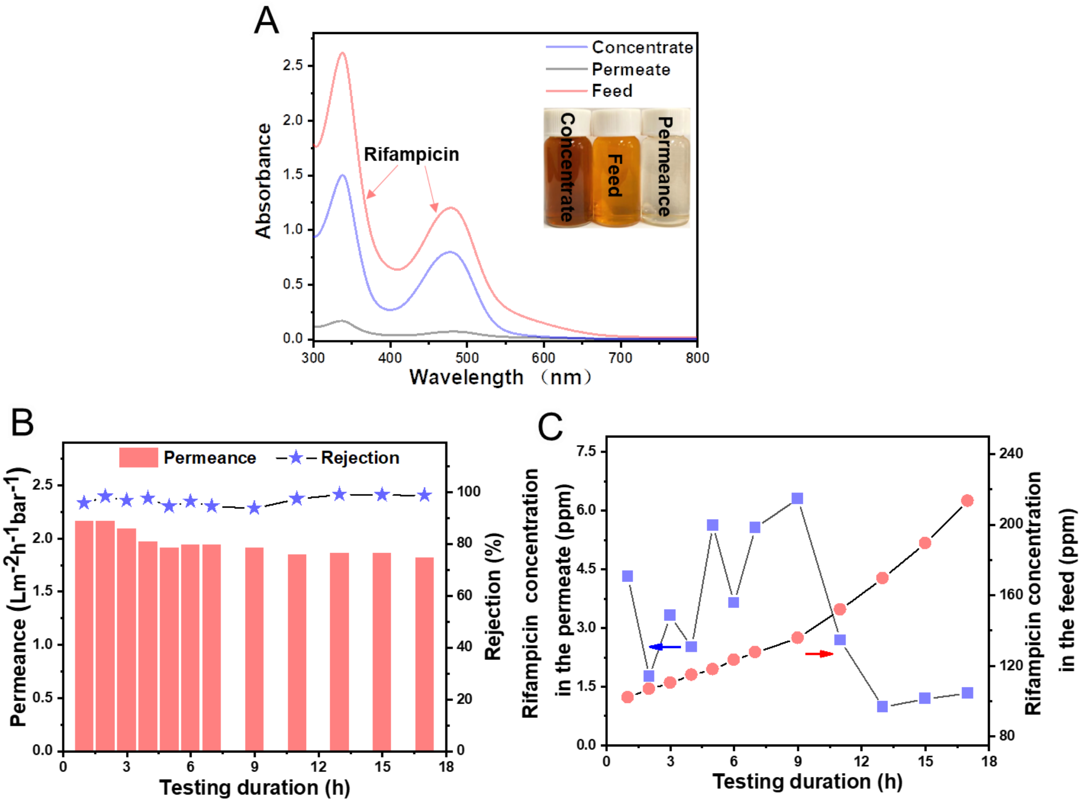

Fig. S33 Concentration experiment of rifampicin. (A) UV-vis absorption spectra of rifampicin in the feed and permeate. (B and C) Separation performance over time of PAR-TN mmembrane as a function of testing duration in concentrating 100 ppm rifampicin in ethanol.

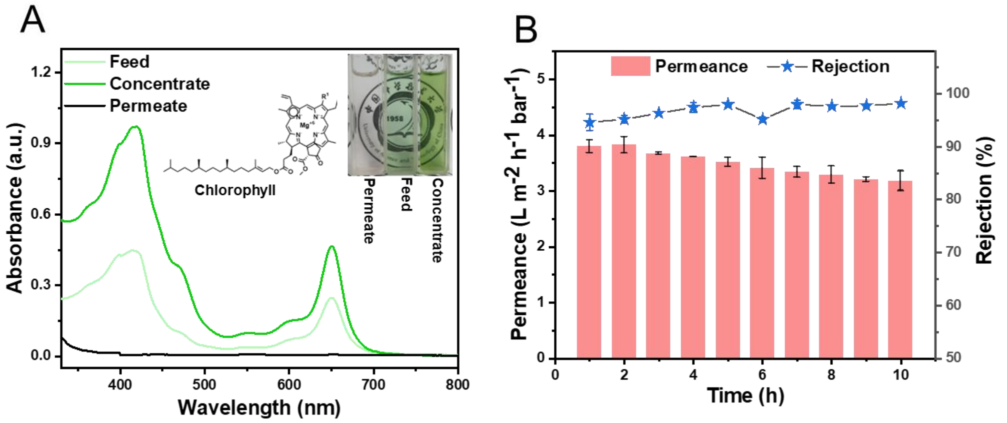

Fig. S34 Concentration experiment of chlorophyll. (A) UV-vis absorption spectra of rifampicin in the feed and permeate. (B) Separation performance over time of PAR-PXN nanofilm as a function of testing duration in concentrating 100 ppm chlorophyll in methanol.

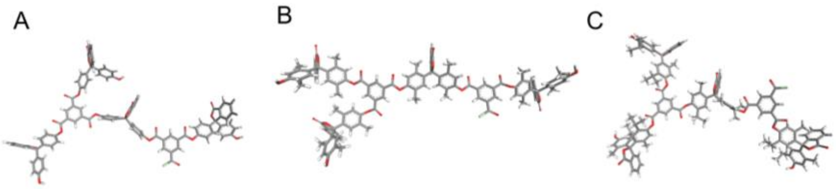

Fig. S35 Molecular model of a segment of polyarylate network containing contorted phenolphthalein monomers. (A) PAR-PN, (B) PAR-PXN, and (C) PAR-TN.

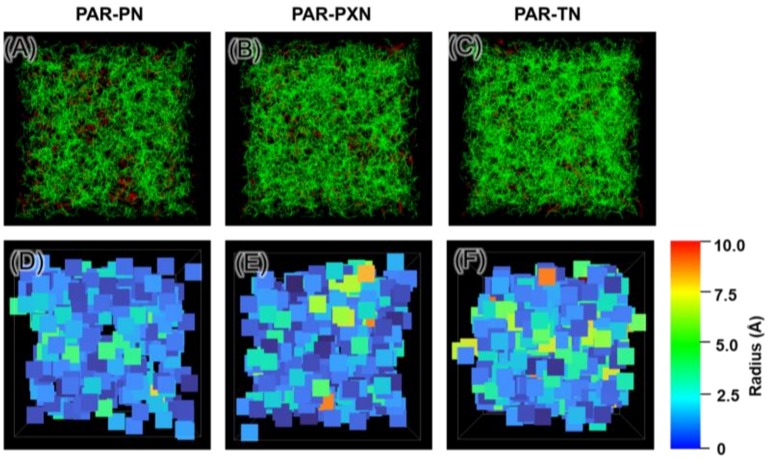

Fig. S36 Structural analysis of amorphous polymer models. (A-C) Interconnected (green) and disconnected (red) voids in polyarylate network with respect to a probe of 0.7 Å radius, which could be distributed across the rigid models. (D-F) The corresponding voids of the polyarylate network colored with respect to the pore radius are shown.

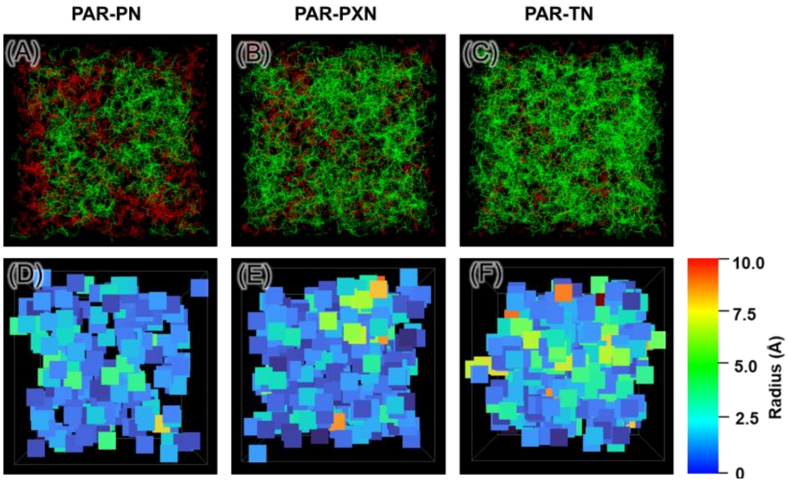

Fig. S37 Structural analysis of amorphous polymer models. (A-C) Interconnected (green) and disconnected (red) voids in polyarylate network with respect to a probe of 0.8 Å radius, which could be distributed across the rigid models. (D-F) The corresponding voids of the polyarylate network colored with respect to the pore radius are shown.

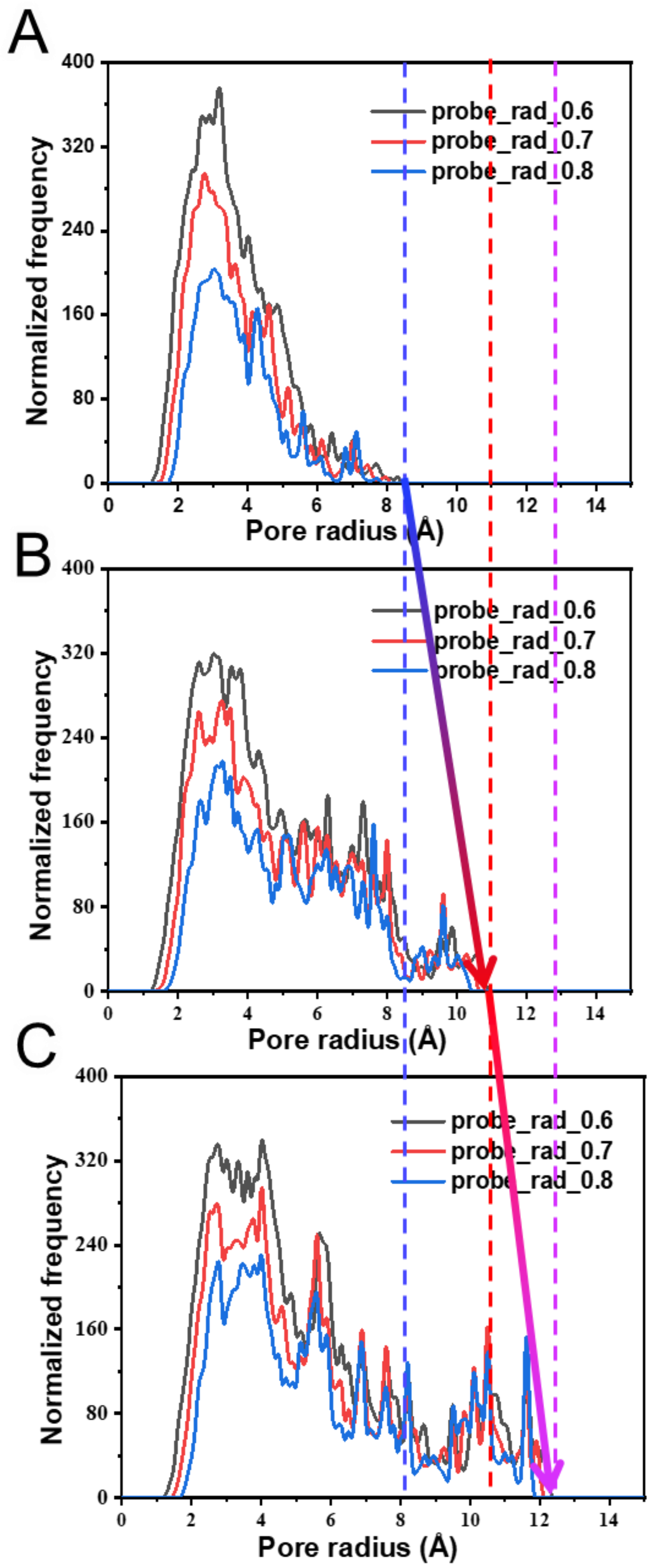

Fig. S38 Simulated aperture distribution. (A) PAR-PN, (B) PAR-PXN, and (C) PAR-TN membranes with probes of 0.6, 0.7, and 0.8 Å, respectively.

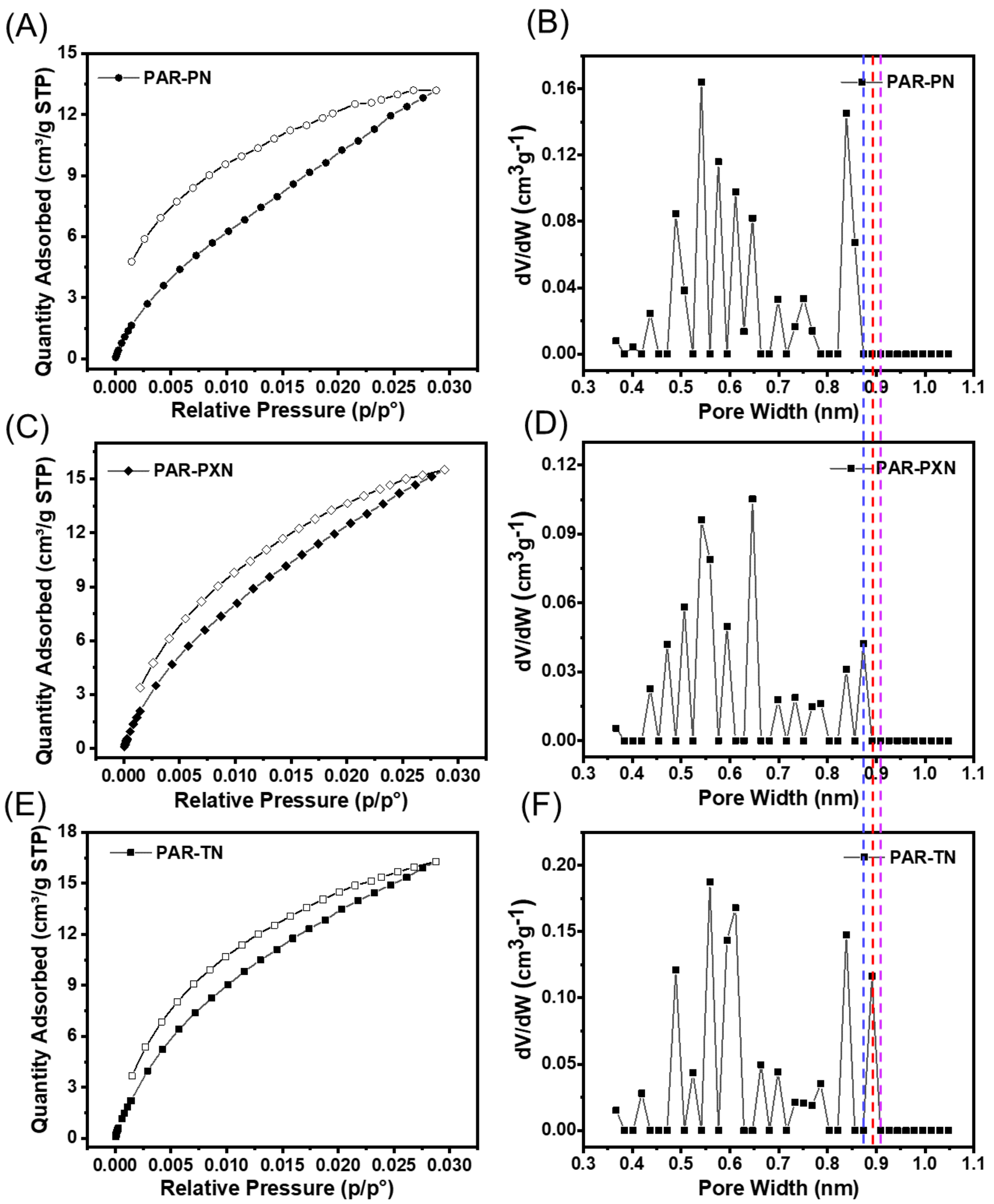

Fig. S39 BET surface area and pore size analysis for different polyarylate nanofilms. CO_2 sorption isotherm curves collected at 273K and corresponding pore-size distribution profiles for (A, B) PAR/TMC-PN, (C, D) PAR/TMC-PXN and (E, F) PAR/TMC-TN.

Table S6. Comparisons of OSN performance of the polyarylate membranes with those of others membranes reported in the state-of-the-art literature.

Membranes	Dye	Molecular weight (g mol^{-1})	Methanol Permeance (L m^{-2} h^{-1} bar^{-1})	Dye rejection (%)	Ref
PAR/TMC-0.1-PN-0.3	Neutral Red	289	7.2	91	This work
PAR/TMC-0.1-PN-0.2	Direct Red 23	814	9.9	93.2	
PAR/TMC-0.3-PXN-0.2	Neutral Red	289	3.9	94.5	
PAR/TMC-0.05-TN-0.2	Direct Red 23	814	7.4	92.5	
PAR/TMC-0.2-TN-0.2	Neutral Red	289	3.3	91.3	
MPDTrip-20	Sudan Orange G	214	9.1	99.1	*(13)*
MPDTMC-20	Sudan Orange G	214	4.8	97.9	*(13)*
PI/ZIF-8/PA	Sunset Yellow	452	8.7	90	*(61)*
PI/LS-MIL101(Cr)/PA	Sunset Yellow	452	8.7	91	*(62)*
PVDF/*t*-Cu-TCPP/PA	Congo red	697	9.2	95	*(63)*
PVDF/*i*-Cu-TCPP/PA	Methylene Blue	320	4.4	96.7	*(64)*
P84/HKUST/PA	Brilliant Blue G	858	9.5	98.8	
Synder Filtration™ NDX	Congo Red	697	2.5	98.6	*(45)*
SC-AA-TPC-0.1	Congo red	697	3.1	94.3	*(45)*
Solsep 030705	Triolein	885	1.4	100	*(65)*
m-XDA-TMC/ANF	Methyl orange	327	2.5	99.6	*(66)*
PARDHAQ	Crystal violet	408	0.6	> 95	*(15)*
PARRES	Crystal violet	408	0.6	> 95	*(15)*
Alumina/PA-ACT	Acid fuchsin	586	2.1	96.2	
XP84/PA-ACT	Acid fuchsin	586	4.4	99.9	

CS-PANI	Polystyrene	230	0.8	97	*(67)*
PPSf/PI	Sudan II	276	2.0	95	*(68)*
PA/P84	Polystyrene	230	1.5	98	*(69)*
PA/PP	Brilliant Blue R	826	0.15	88	*(70)*
PA/PSf	Bromothymol lue	624	2	> 90	*(71)*
PA/cross-linked P84 PI	styrene oligomers	236	1.5	98	*(72)*
(PIM-1/PEi)/PAN	styrene oligomers	236	3.6	90	*(73)*
Alumina/PA	Acid fuchsin	586	6.3	96.5	*(52)*
Freestanding cis-CON	Indigo carmine	446	1.9	94.7	*(74)*
Freestanding trans-CON	Brilliant Blue R	826	1.7	98.7	*(74)*
TPC-N4	Brilliant Blue R	826	6.2	99.7	*(75)*
IPC-N4	Brilliant Blue R	826	5.5	99.1	*(75)*

REFERENCES AND NOTES

1. Y. Cui, T.-S. Chung, Solvent recovery via organic solvent pressure assisted osmosis. *Ind. Eng. Chem. Res.* **58**, 4970–4978 (2019).

2. P. Vandezande, L. E. M. Gevers, I. F. J. Vankelecom, Solvent resistant nanofiltration: Separating on a molecular level. *Chem. Soc. Rev.* **37**, 365–405 (2008).

3. Y. Cui, T.-S. Chung, Pharmaceutical concentration using organic solvent forward osmosis for solvent recovery. *Nat. Commun.* **9**, 1426 (2018).

4. P. Marchetti, M. F. Jimenez Solomon, G. Szekely, A. G. Livingston, Molecular separation with organic solvent nanofiltration: A critical review. *Chem. Rev.* **114**, 10735–10806 (2014).

5. D. S. Sholl, R. P. Lively, Seven chemical separations to change the world. *Nature* **532**, 435–437 (2016).

6. K. Wang, X. Wang, B. Januszewski, Y. Liu, D. Li, R. Fu, M. Elimelech, X. Huang, Tailored design of nanofiltration membranes for water treatment based on synthesis-property-performance relationships. *Chem. Soc. Rev.* **51**, 672–719 (2022).

7. W. J. Koros, C. Zhang, Materials for next-generation molecularly selective synthetic membranes. *Nat. Mater.* **16**, 289–297 (2017).

8. G. M. Shi, Y. Feng, B. Li, H. M. Tham, J.-Y. Lai, T.-S. Chung, Recent progress of organic solvent nanofiltration membranes. *Prog. Polym. Sci.* **123**, 101470 (2021).

9. Y. Yin, S. Liu, J. Zhou, Y. Peng, E. Wang, L. Han, B. Su, Polyamide thin film nanocomposite with in-situ co-constructed COFs for organic solvent nanofiltration. *J. Membr. Sci.* **686**, 122000 (2023).

10. R. P. Lively, D. S. Sholl, From water to organics in membrane separations. *Nat. Mater.* **16**, 276–279 (2017).

11. E. M. P. Rundquist, C. J. Pink, A. G. Livingston, Organic solvent nanofiltration: A potential alternative to distillation for solvent recovery from crystallisation mother liquors. *Green Chem.* **14**, 2197–2205 (2012).

12. J. Zhu, S. Yuan, J. Wang, Y. Zhang, M. Tian, B. Van der Bruggen, Microporous organic polymer-based membranes for ultrafast molecular separations. *Prog. Polym. Sci.* **110**, 101308 (2020).

13. Z. Ali, B. S. Ghanem, Y. Wang, F. Pacheco, W. Ogieglo, H. Vovusha, G. Genduso, U. Schwingenschlogl, Y. Han, I. Pinnau, Finely tuned submicroporous thin-film molecular sieve membranes for highly efficient fluid separations. *Adv. Mater.* **32**, e2001132 (2020).

14. S. L. Li, G. Chang, Y. Huang, K. Kinooka, Y. Chen, W. Fu, G. Gong, T. Yoshioka, N. B. McKeown, Y. Hu, 2,2'-Biphenol-based ultrathin microporous nanofilms for highly efficient molecular sieving separation. *Angew. Chem. Int. Ed. Engl.* **61**, e202212816 (2022).

15. M. F. Jimenez-Solomon, Q. Song, K. E. Jelfs, M. Munoz-Ibanez, A. G. Livingston, Polymer nanofilms with enhanced microporosity by interfacial polymerization. *Nat. Mater.* **15**, 760–767 (2016).

16. N. Gao, S. Zhang, Phenolphthalein-based cardo poly(arylene ether sulfone): Preparation and application to separation membranes. *J. Appl. Polym. Sci.* **128**, 1–12 (2013).

17. H. Jiang, Y. Qi, T. Chen, S. Bo, M. Ding, L. Gao, J. Xu, T. He, Y. Zhang, Synthesis, structure and ring-opening polymerization of phenolphthalein macrocyclic polyarylates. *Macromol. Chem. Phys.* **201**, 2385–2393 (2000).

18. A. J. Eherer, C. A. S. Ana, J. Porter, J. S. Fordtran, Effect of psyllium, calcium polycarbophil, and wheat bran on secretory diarrhea induced by phenolphthalein. *Gastroenterology* **104**, 1007–1012 (1993).

19. J. Zhao, Z. Wang, H. Wang, G. Zhou, H. Nie, High-expanded foams based on novel long-chain branched poly(aryl ether ketone) via $ScCO_2$ foaming method. *Polymer* **165**, 124–132 (2019).

20. R. Xu, L. He, L. Li, M. Hou, Y. Wang, B. Zhang, C. Liang, T. Wang, Ultraselective carbon molecular sieve membrane for hydrogen purification. *J. Energy Chem.* **50**, 16–24 (2020).

21. A. Mohanty, Y. E. Song, J. R. Kim, N. Kim, H.-J. Paik, Phenolphthalein anilide based poly(ether sulfone) block copolymers containing quaternary ammonium and imidazolium cations: Anion exchange membrane materials for microbial fuel cell. *Membranes* **11**, 454 (2021).

22. D. Ren, Y.-H. Li, S.-P. Ren, T.-Y. Liu, X.-L. Wang, Microporous polyarylate membrane with nitrogen-containing heterocycles to enhance separation performance for organic solvent nanofiltration. *J. Membr. Sci.* **610**, 118295 (2020).

23. J. Zhao, Z. Wang, H. Wang, G. Zhou, Novel poly(aryl ether nitrile ketone) foams and the influence of copolymer structure on the foaming result. *Polym. Int.* **67**, 1410–1418 (2018).

24. R. Xu, L. Li, Y. Wang, M. Hou, Z. Pan, C. Song, T. Wang, Thermal crosslinking membrane with enhanced CO_2 separation performance derived from nitrile-containing phenolphthalein-based poly(arylene eher ketone). *J. Membr. Sci.* **637**, 119634 (2021).

25. W. Y. Liu, T. L. Chen, J. P. Xu, Gas permeation behavior of phenolphthalein based heat-resistant polymers PEK-C and PES-C. *J. Membr. Sci.* **53**, 203–213 (1990).

26. R. Xu, M. Hou, Y. Wang, L. Li, Z. Pan, C. Song, T. Wang, High-performance carbon molecular sieve membrane for C_2H_4/C_2H_6 separation: Molecular insight into the structure-property relationships. *Carbon* **201**, 24–36 (2023).

27. M. Zhao, C. Zhang, Y. Weng, P. Li, Synergistic improvement of CO_2/CH_4 separation performance of phenolphthalein-based polyimide membranes by thermal decomposition and thermal-oxidative crosslinking. *Polymer* **263**, 125528 (2022).

28. P. Du, Z. Wang, T. Zhang, C. H. Lau, S. Liu, P. Li, Crosslinked thermally rearranged polybenzoxazole derived from phenolphthalein-based polyimide for gas separation. *J. Membr. Sci.* **662**, 120934 (2022).

29. X. Yan, H. Wan, X. Xing, J. Yang, G. Yan, G. Zhang, High permeance nanofiltration membrane for harsh organic solvent based on spiral-ring polyesters. *J. Membr. Sci.* **687**, 122035 (2023).

30. B. Liang, H. Wang, X. Shi, B. Shen, X. He, Z. A. Ghazi, N. A. Khan, H. Sin, A. M. Khattak, L. Li, Z. Tang, Microporous membranes comprising conjugated polymers with rigid backbones enable ultrafast organic-solvent nanofiltration. *Nat. Chem.* **10**, 961–967 (2018).

31. Y. Bai, B. Liu, J. Li, M. Li, Z. Yao, L. Dong, D. Rao, P. Zhang, X. Cao, L. F. Villalobos, C. Zhang, Q. F. An, M. Elimelech, Microstructure optimization of bioderived polyester nanofilms for antibiotic desalination via nanofiltration. *Sci. Adv.* **9**, eadg6134 (2023).

32. D. W. Burke, Z. Jiang, A. G. Livingston, W. R. Dichtel, 2D covalent organic framework membranes for liquid-phase molecular separations: State of the field, common pitfalls, and future opportunities. *Adv. Mater.* **36**, e2300525 (2023).

33. J. Rivnay, S. C. Mannsfeld, C. E. Miller, A. Salleo, M. F. Toney, Quantitative determination of organic semiconductor microstructure from the molecular to device scale. *Chem. Rev.* **112**, 5488–5519 (2012).

34. D. Liang, C. Dong, L. Cai, Z. Su, J. Zang, C. Wang, X. Wang, Y. Zou, Y. Li, L. Chen, L. Zhang, Z. Hong, A. El-Shaer, Z. K. Wang, X. Gao, B. Sun, Unveiling crystal orientation in quasi-2D perovskite films by in situ GIWAXS for high-performance photovoltaics. *Small* **17**, e2100972 (2021).

35. T. Y. Cath, S. Gormly, E. G. Beaudry, M. T. Flynn, V. D. Adams, A. E. Childress, Membrane contactor processes for wastewater reclamation in space. *J. Membr. Sci.* **257**, 85–98 (2005).

36. Z. Tan, S. Chen, X. Peng, L. Zhang, C. Gao, Polyamide membranes with nanoscale turing structures for water purification. *Science* **360**, 518–521 (2018).

37. S. Karan, Z. Jiang, A. G. Livingston, Sub-10 nm polyamide nanofilms with ultrafast solvent transport for molecular separation. *Science* **348**, 1347–1351 (2015).

38. Y. Zhang, Y. Wan, G. Pan, X. Wei, Y. Li, H. Shi, Y. Liu, Preparation of high performance polyamide membrane by surface modification method for desalination. *J. Membr. Sci.* **573**, 11–20 (2019).

39. L. Liu, C. Yin, Y. Li, H. Yang, Y. Du, Y. Wang, Alkyl-engineered hydrophobic channels in covalent organic frameworks toward fast organic solvent nanofiltration. *Ind. Eng. Chem. Res.* **62**, 21304–21310 (2023).

40. J. Liu, G. Han, D. Zhao, K. Lu, J. Gao, T. S. Chung, Self-standing and flexible covalent organic ramework (COF) membranes for molecular separation. *Sci. Adv.* **6**, eabb1110 (2020).

41. C. Kim, D. Y. Koh, Y. J. Lee, J. Choi, H. S. Cho, M. Choi, Bottom-up synthesis of two-dimensional carbon with vertically aligned ordered micropores for ultrafast nanofiltration. *Sci. Adv.* **9**, eade7871 (2023).

42. X. Shi, Z. Zhang, C. Yin, X. Zhang, J. Long, Z. Zhang, Y. Wang, Design of three-dimensional covalent organic framework membranes for fast and robust organic solvent nanofiltration. *Angew. Chem. Int. Ed. Engl.* **61**, e202207559 (2022).

43. L. Cao, I. C. Chen, Z. Li, X. Liu, M. Mubashir, R. A. Nuaimi, Z. Lai, Switchable Na^+ and K^+ selectivity in an amino acid functionalized 2D covalent organic framework membrane. *Nat. Commun.* **13**, 7894 (2022).

44. X. Jing, M. Zhang, Z. Mu, P. Shao, Y. Zhu, J. Li, B. Wang, X. Feng, Gradient channel segmentation in covalent organic framework membranes with highly oriented nanochannels. *J. Am. Chem. Soc.* **145**, 21077–21085 (2023).

45. Z. Jiang, R. Dong, A. M. Evans, N. Biere, M. A. Ebrahim, S. Li, D. Anselmetti, W. R. Dichtel, A. G. Livingston, Aligned macrocycle pores in ultrathin films for accurate molecular sieving. *Nature* **609**, 58–64 (2022).

46. H. Fan, J. He, M. Heiranian, W. Pan, Y. Li, M. Elimelech, The physical basis for solvent flow in organic solvent nanofiltration. *Sci. Adv.* **10**, eado4332 (2024).

47. H. Sun, X. Li, N. Wang, Q.-F. An, Defect engineering on zeolitic imidazolate framework membrane via thermal annealing for organic solvent nanofiltration. *Sep. Purif. Technol.* **310**, 123220 (2023).

48. S. Kumar, N. Alqadhi, J. Hu, G. Szekely, Swift fabrication of thin-film composite membranes with azine-linked covalent organic framework for high-temperature organic solvent nanofiltration. *J. Membr. Sci.* **691**, 122257 (2024).

49. J. Cavalcante, D. G. Oldal, M. V. Peskov, A. K. Beke, R. Hardian, U. Schwingenschlögl, G. Szekely, Biobased interpenetrating polymer network membranes for sustainable molecular sieving. *ACS Nano* **18**, 7433–7443 (2024).

50. S. Plimpton, Fast parallel algorithms for short-range molecular dynamics. *J. Comput. Phys.* **117**, 1–19 (1995).

51. T. F. Willems, C. H. Rycroft, M. Kazi, J. C. Meza, M. Haranczyk, Algorithms and tools for high-throughput geometry-based analysis of crystalline porous materials. *Microporous Mesoporous Mater.* **149**, 134–141 (2012).

52. Y. Zhang, X. Cheng, X. Jiang, J. J. Urban, C. H. Lau, S. Liu, L. Shao, Robust natural nanocomposites realizing unprecedented ultrafast precise molecular separations. *Mater. Today* **36**, 40–47 (2020).

53. L. Shen, Q. Shi, S. P. Zhang, J. Gao, D. C. Cheng, M. Yi, R. Y. Song, L. D. Wang, J. W. Jiang, R. Karnik, S. Zhang, Highly porous nanofiber-supported monolayer graphene membranes for ultrafast organic solvent nanofiltration. *Sci. Adv.* **7**, eabg6263 (2021).

54. L. Zhang, M. Zhang, G. Liu, W. Jin, X. Li, Fungal cell wall-graphene oxide microcomposite membrane for organic solvent nanofiltration. *Adv. Funct. Mater.* **31**, 2100110 (2021).

55. L. Nie, K. Goh, Y. Wang, J. Lee, Y. Huang, H. E. Karahan, K. Zhou, M. D. Guiver, T.-H. Bae, Realizing small-flake graphene oxide membranes for ultrafast size-dependent organic solvent nanofiltration. *Sci. Adv.* **6**, eaaz9184 (2020).

56. P. He, S. Zhao, C. Mao, Y. Wang, G. Ma, Z. Wang, J. Wang, In-situ growth of double-layered polyaniline composite membrane for organic solvent nanofiltration. *Chem. Eng. J.* **420**, 129338 (2021).

57. M.-B. Wu, F. Yang, J. Yang, Q. Zhong, V. Körstgen, P. Yang, P. Müller-Buschbaum, Z.-K. Xu, Lysozyme membranes promoted by hydrophobic substrates for ultrafast and precise organic solvent nanofiltration. *Nano Lett.* **20**, 8760–8767 (2020).

58. X. He, H. Sin, B. Liang, Z. A. Ghazi, A. M. Khattak, N. A. Khan, H. R. Alanagh, L. Li, X. Lu, Z. Tang, Controlling the selectivity of conjugated microporous polymer membrane for efficient organic solvent nanofiltration. *Adv. Funct. Mater.* **29**, 1900134 (2019).

59. T. Huang, B. A. Moosa, P. Hoang, J. Liu, S. Chisca, G. Zhang, M. AlYami, N. M. Khashab, S. P. Nunes, Molecularly-porous ultrathin membranes for highly selective organic solvent nanofiltration. *Nat. Commun.* **11**, 5882 (2020).

60. L. Sarango, L. Paseta, M. Navarro, B. Zornoza, J. Coronas, Controlled deposition of mofs by dip-coating in thin film nanocomposite membranes for organic solvent nanofiltration. *J. Ind. Eng. Chem.* **59**, 8–16 (2018).

61. M. Navarro, J. Benito, L. Paseta, I. Gascon, J. Coronas, C. Tellez, Thin-film nanocomposite membrane with the minimum amount of MOF by the Langmuir-Schaefer technique for nanofiltration. *ACS Appl. Mater. Interfaces* **10**, 1278–1287 (2018).

62. Yao Ayan, Hua Dan, Hong Yiping, Pan Junyang, Cheng Xi, Tan Kok Bing, Z. Guowu, Using Cu-TCPP nanosheets as interlayers for high-performance organic solvent nanofiltration membranes. *ACS Appl. Nano Mater.* **5**, 18718–18729 (2022).

63. A. Yao, D. Hua, Z. F. Gao, J. Pan, A.-R. Ibrahim, D. Zheng, Y. Hong, Y. Liu, G. Zhan, Fabrication of organic solvent nanofiltration membrane using commercial PVDF substrate via interfacial polymerization on top of metal-organic frameworks interlayer. *J. Membr. Sci.* **652**, 120465 (2022).

64. R. Othman, A. W. Mohammad, M. Ismail, J. Salimon, Application of polymeric solvent resistant nanofiltration membranes for biodiesel production. *J. Membr. Sci.* **348**, 287–297 (2010).

65. Y. Li, S. Li, J. Zhu, A. Volodine, B. Van der Bruggen, Controllable synthesis of a chemically stable molecular sieving nanofilm for highly efficient organic solvent nanofiltration. *Chem. Sci.* **11**, 4263–4271 (2020).

66. X. X. Loh, M. Sairam, A. Bismarck, J. H. G. Steinke, A. G. Livingston, K. Li, Crosslinked integrally skinned asymmetric polyaniline membranes for use in organic solvents. *J. Membr. Sci.* **326**, 635–642 (2009).

67. J. C. Jansen, S. Darvishmanesh, F. Tasselli, F. Bazzarelli, P. Bernardo, E. Tocci, K. Friess, A. Randova, E. Drioli, B. Van der Bruggen, Influence of the blend composition on the properties and separation performance of novel solvent resistant polyphenylsulfone/polyimide nanofiltration membranes. *J. Membr. Sci.* **447**, 107–118 (2013).

68. M. F. Jimenez Solomon, Y. Bhole, A. G. Livingston, High flux hydrophobic membranes for organic solvent nanofiltration (OSN)—Interfacial polymerization, surface modification and solvent activation. *J. Membr. Sci.* **434**, 193–203 (2013).

69. P. B. Kosaraju, K. K. Sirkar, Interfacially polymerized thin film composite membranes on microporous polypropylene supports for solvent-resistant nanofiltration. *J. Membr. Sci.* **321**, 155–161 (2008).

70. M. Peyravi, A. Rahimpour, M. Jahanshahi, Thin film composite membranes with modified polysulfone supports for organic solvent nanofiltration. *J. Membr. Sci.* **423-424**, 225–237 (2012).

71. M. F. Jimenez Solomon, Y. Bhole, A. G. Livingston, High flux membranes for organic solvent nanofiltration (OSN)—Interfacial polymerization with solvent activation. *J. Membr. Sci.* **423**, 371–382 (2012).

72. D. Fritsch, P. Merten, K. Heinrich, M. Lazar, M. Priske, High performance organic solvent nanofiltration membranes: Development and thorough testing of thin film composite membranes made of polymers of intrinsic microporosity (PIMs). *J. Membr. Sci.* **401-402**, 222–231 (2012).

73. J. T. Liu, S. F. Wang, T. F. Huang, P. Manchanda, E. Abou-Hamad, S. P. Nunes, Smart covalent organic networks (CONs) with "on-off-on" light-switchable pores for molecular separation. *Sci. Adv.* **6**, eabb3188 (2020).

74. A. Yao, J. Du, Q. Sun, L. Liu, Z. Song, W. He, J. Liu, Flexible covalent organic network with ordered honeycomb nanoarchitecture for molecular separations. *ACS Nano* **17**, 22916–22927 (2023).
